# Precise and efficient C-to-U RNA base editing with SNAP-CDAR-S

**DOI:** 10.1093/nar/gkad598

**Published:** 2023-07-18

**Authors:** Ngadhnjim Latifi, Aline Maria Mack, Irem Tellioglu, Salvatore Di Giorgio, Thorsten Stafforst

**Affiliations:** Interfaculty Institute of Biochemistry, University of Tübingen, Auf der Morgenstelle 15, 72076 Tübingen, Germany; Interfaculty Institute of Biochemistry, University of Tübingen, Auf der Morgenstelle 15, 72076 Tübingen, Germany; Division of Immune Diversity (D150), German Cancer Research Center (DKFZ), 69120 Heidelberg, Germany; Faculty of Engineering, University of Heidelberg, 69120 Heidelberg, Germany; Division of Immune Diversity (D150), German Cancer Research Center (DKFZ), 69120 Heidelberg, Germany; Interfaculty Institute of Biochemistry, University of Tübingen, Auf der Morgenstelle 15, 72076 Tübingen, Germany; Gene and RNA Therapy Center (GRTC), Faculty of Medicine University Tuebingen, Germany

## Abstract

Site-directed RNA base editing enables the transient and dosable change of genetic information and represents a recent strategy to manipulate cellular processes, paving ways to novel therapeutic modalities. While tools to introduce adenosine-to-inosine changes have been explored quite intensively, the engineering of precise and programmable tools for cytidine-to-uridine editing is somewhat lacking behind. Here we demonstrate that the cytidine deaminase domain evolved from the ADAR2 adenosine deaminase, taken from the RESCUE-S tool, provides very efficient and highly programmable editing when changing the RNA targeting mechanism from Cas13-based to SNAP-tag-based. Optimization of the guide RNA chemistry further allowed to dramatically improve editing yields in the difficult-to-edit 5′-CCN sequence context thus improving the substrate scope of the tool. Regarding editing efficiency, SNAP-CDAR-S outcompeted the RESCUE-S tool clearly on all tested targets, and was highly superior in perturbing the β-catenin pathway. NGS analysis showed similar, moderate global off-target A-to-I and C-to-U editing for both tools.

## Graphical Abstract

**Graphical Abstract:**
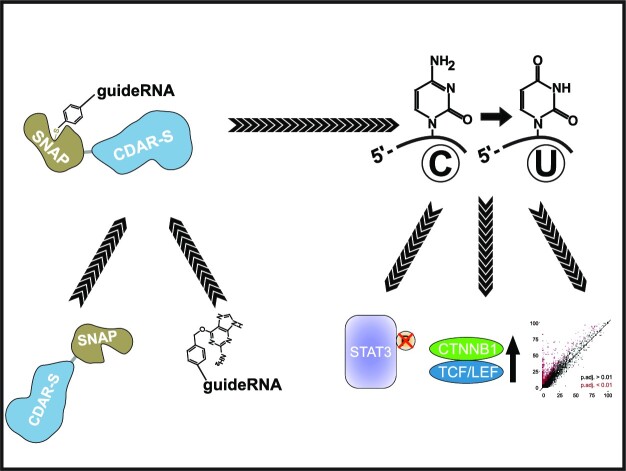

## INTRODUCTION

Cytidine (C) deamination yielding uridine (U) is a well-known posttranscriptional reaction that diversifies genetic information at the RNA level (1). The enzymatic base conversion is carried out by hydrolases/deaminases belonging to the class of AID/APOBEC proteins, of which some are specific for RNA, while others can use both RNA and DNA, or only DNA as substrates. The first C-to-U RNA editing enzyme described was APOBEC1 (APO1) (2), which catalyzes the switch from the long ApoB100 to the short ApoB48 isoform by rewriting a glutamine codon (5′-CAA) into a STOP codon (5′-UAA) in the enterocytes of the small intestine (3). Later, single-strand RNA editing activity of further members of the APOBEC family, including APOBEC3A and 3G, was discovered. However, the biological function and targets of their RNA editing activity remained unclear to some extent (1). C-to-U RNA editing activity is typically found only in a sub-set of tissues, like small intestine and liver for APO1, or monocytes and macrophages for A3A, but can be up- and down-regulated in various pathologic situations and play a role in tumor evolution (4), for example (5). AID/APOBEC enzymes are often recruited to their targets by the help of auxiliary proteins, e.g. RBM47 (6) and A1CF (7) for APO1, and have a very strong and thus limiting preference for specific di-nucleotides as editing substrates (1). Highly edited substrates, like the Glutamine-to-STOP site in the ApoB transcript, are placed in specific secondary structures that assist the recruitment and activity of the deaminase (8,9).

**Figure 1.**
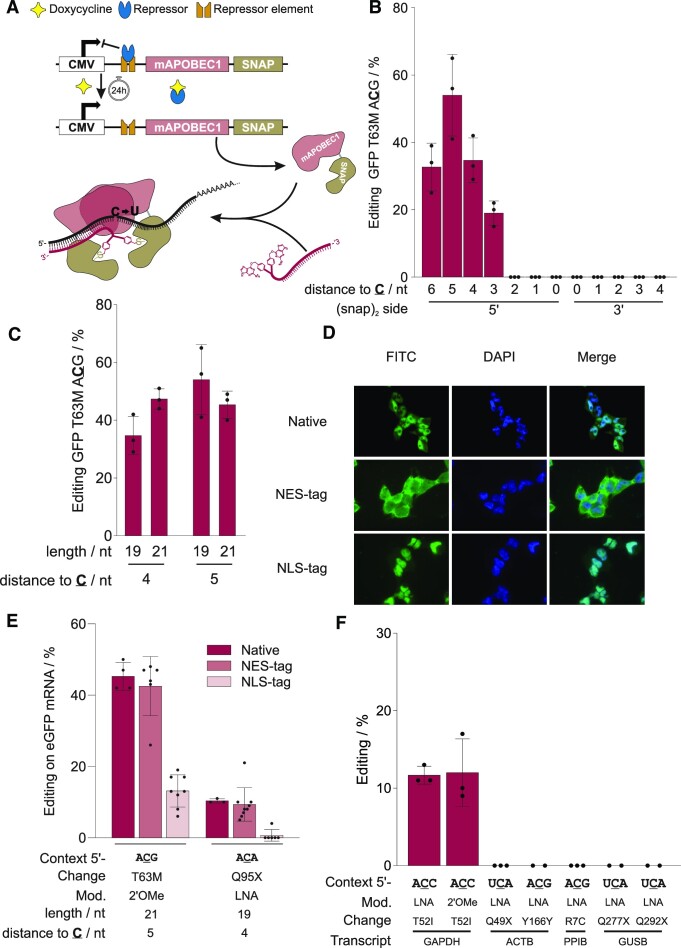
Properties of the APO1S tool. (**A**) Scheme of the doxycycline-inducible APO1S tool and the guide RNA-dependent editing reaction. (**B**) Dependency of editing yield of the T63M site (5′-ACG) in the eGFP reporter gene on the positioning of the guide RNA (19 nucleotides length, fully 2′OMe modified) relative to the target site (‘C’). (**C**) Fine-tuning of guide RNA length (19, 21 nt) and positioning (4 or 5 nt 5′ to the target cytidine) for optimal editing performance. (**D**) Analysis of APO1S transgene expression and localization by fluorescence microscopy. The native mApobec1 sequence was either amended with an NES or NLS tag, as indicated, leading to cytosolic or nuclear localization of the APO1S protein, which was stained with SNAP-tag-reactive BG-FITC (green channel). (**E**) Effect of editase localization on editing of two sites in an eGFP reporter with the respective best performing guideRNA design. (**F**) Editing performance of guide RNAs (21 nt, position 5) with different backbone chemistries (2′-OMe, LNA) inducing the indicated amino acid changes at the respective endogenous transcripts demonstrating limited programmability of the tool. Data in (B), (C), (E) and (F) are shown as the mean ± s.d. of *N* ≥ 2 independent experiments as represented by individual data points.

Targeted RNA base editing aims at harnessing C-to-U and A(denosine)-to-I(nosine) editing activity for the rewriting of genetic information, including the substitution of amino acids and the formation (C-to-U) or removal (A-to-I) of premature STOP codons (10). The approach opens novel avenues for drug discovery, promising to bypass technical and ethical issues related to genome editing (10). In this field, our lab contributed an RNA-targeting platform based on fusion proteins of the self-labeling SNAP-tag (11) (Figure 1A) (12). Initially, we engineered a programmable A-to-I RNA base editor by fusing the SNAP-tag (11) with the catalytic domain of the RNA editing enzyme ADAR (adenosine deaminase acting on RNA) (13,14). In these fusions, the SNAP-tag exploits its self-labeling activity to covalently tether to a guideRNA in a defined 1:1 stoichiometry by recognizing a benzylguanine (BG) moiety, the so-called self-labeling moiety, at the guideRNA. According to simple Watson-Crick base-pairing rules, the guideRNA addresses the editing of one specific adenosine residue in a selected transcript with high efficiency, broad codon scope, and very good precision (14). Competing RNA-targeting platforms have been developed based on Cas proteins (15), or trans-tethering approaches (16). While each approach has its specific strength and weakness (10,12), a clear advantage of the SNAP-tag approach is its human origin, its small size, the ease of transfecting of the chemically stabilized guideRNA(s) (14), the possibility of concurrent editing (14), and the ready inclusion of small molecule (17) and photo control (18,19). Furthermore, we have recently shown the concurrent and fully orthogonal usage of two independent RNA editing effectors by complementing a SNAP-tagged C-to-U editing effector with a HALO-tagged A-to-I editing tool within the same cell (20). In the latter study, we exploited the C-to-U deaminase domain from murine APO1. Other labs have developed C-to-U RNA base editing effectors based on human APO1 or APO3A. In the first example, RNA-targeting was based on the trans-tethering approach with the MS2/MCP system (21). In the latter, the dCas13 platform was applied (22). However, none of these approaches is yet working optimally. Our approach with SNAP-tagged APO1 (20) gave low editing yields on endogenous targets and its programmability, which is the addressing of any given target cytidine with a guide RNA, was somehow limited due to the strong requirement (8) for APO1 substrates to be located in specific secondary structures. While this was better solved for the A3A target (22), this tool suffers from the strong substrate codon preference for 5′-UC. An exciting alternative came from the engineering of an artificial C-to-U editing enzyme. Specifically, laboratory evolution was used to engineer the A-to-I deaminase domain of the hyperactive E488Q mutant (23) of the ancestor ADAR2 into a C-to-U editing enzyme (24). With a dCas13-based RNA-targeting mechanism the so-called RESCUE tool was steered to its target RNAs. While the programmability was good, the editing yields remained moderate for 5′-WC (W = A or U) codons and low for 5′-CC, whereas 5′-GC codons were hardly editable (24), mirroring the well-known codon preference (25) of ADAR2. Furthermore, the RESCUE tool retained notable A-to-I off-target editing beside C-to-U off-target editing. A high-fidelity variant, RESCUE-S, was developed (23), that carried an additional point mutation. However, the point mutation lowered both, the C-to-U on-target and the A-to-I off-target editing yields.

Here, we now show that the high-fidelity cytidine deaminase acting on RNA (CDAR) domain from the RESCUE-S tool works very well when we replace the dCas13 domain with a SNAP-tag for RNA targeting. In particular on endogenous transcripts, the SNAP-CDAR-S outcompetes the RESCUE-S tool clearly and achieves moderate to good editing yields for all 5′-HC codons (H = C, A, U) under very good control of A-to-I and C-to-U bystander editing.

## MATERIALS AND METHODS

### Generation of guideRNAs

guideRNAs (gRNA) were designed and purchased from Eurogentec or Sigma-Aldrich with a 5′-C6-Amino linker or a 3′-C7-Amino linker referred to as NH_2_-gRNA. snap/(snap)_2_-gRNA synthesis was carried out as previously described (20). Briefly, snap-linker was pre-activated with EDCI for 60 min at 30°C and (snap)_2_-linker was pre-activated with DIC for 4 h at 45°C or over-night at 37°C. Coupling of snap-linker to gRNA was carried out at 30°C for 90 min and coupling of (snap)_2_-Linker was carried out at 37°C for 2 h. Purification of gRNA was carried out by 5M urea PAGE and subsequent ethanol precipitation. Concentration and purity were determined by NanoDrop™ 2000/2000c Spectrophotometers (ThermoFisher Scientific). A detailed protocol is also given in the Supplementary Information (pages 3–4).

### Generation of stable cell lines and editing

All transgenic cell lines were created from parental HEK 293 Flp-In™ T-REx™ cells (Invitrogen). Parental cells were cultivated in Dulbecco's modified Eagle's medium (DMEM; Life Technologies) with fetal bovine serum (FBS; Life Technologies, 10% final conc.), 100 μg/ml Zeocin (Z; ThermoFisher) and 15 μg/ml Blasticidin (B; Blasticidin S Hydrochloride, Carl Roth) 37°C and 5% CO_2_. For generation of transgenic cell lines, 1.6 × 10^6^ of parental cells were transfected with 4 μg of a 9:1 ratio of pOG44 Flp-Recombinase expression vector (Invitrogen) and pcDNA™5/FRT Mammalian Expression Vector (Invitrogen) bearing the transgene of interest using Lipofectamine™ 2000 (Life Technologies). For successful generation of transgenic cells, cells were selected for two weeks with DMEM, FBS (10%), B, 100 μg/ml Hygromycin (H; Carl Roth). Cells were then kept in DMEM, FBS (10%), B, H at 37°C and 5% CO_2_. For SNAP-editase expressing cells, 3 × 10^5^ cells were seeded in DMEM, FBS (10%) and doxycycline (Dox; PanReacAppliChem, 10 ng/ml final conc.) in a 24-well format for transgene induction. 24 h after seeding cells were transfected with 300 ng of pcDNA3.1 expressing eGFP or pDNA expressing APOE4 (Addgene, #87087) with 0.9 μl of Lipofectamine™ 2000 (Life Technologies) per 300 ng of pDNA. 24h post-transfection, 8 × 10^4^ cells were transfected with 5 pmol (unless differently stated) of snap/(snap)_2_-gRNA with 0.75 μl of Lipofectamine™ 2000 per 5 pmol of gRNA. Unless differently stated, endpoint was 48 h post gRNA transfection. If endogenous transcripts were targeted, pDNA expression was omitted. For RESCUE-S expressing cells, 2 × 10^4^ cells were seeded in a 96-well format in DMEM, FBS (10%) and doxycycline (10 ng/ml final conc.) for transgene induction. 24 h later, cells were transfected similarly to as previously described. Briefly, cells were transfected with 300 ng of gRNA expressing pDNA and if applicable 40 ng of reporter pDNA with 0.5 μl of Lipofectamine™ 2000. Unless differently stated, endpoint was 48 h post transfection.

### Editing yield analysis

At endpoint, cells were lysed in 50 μl per well (96-well format) RLT buffer (Qiagen). Total RNA was isolated using the Monarch^®^ RNA Cleanup Kit 10 μg (New England BioLabs) following manufacturer's instructions. Target sites were amplified using either One Step RT-PCR Kit (BiotechRabbit) or One*Taq^®^* One-Step RT-PCR Kit (New England BioLabs) and the appropriate primers. Sanger sequencing was performed by Microsynth or Eurofins. Editing yield was determined as the ratio of peak heights at target sites in the chromatogram of samples.

### Microscopy

5 × 10^4^ Flp-In™ T-Rex™ cells expressing SNAP-editases were seeded on glass cover slips coated with poly-(d)-lysine hydrobromide (Sigma-Aldrich, 0.1 mg/ml in H_2_O) in DMEM, FBS (10%), B, H with or without doxycycline (10 ng/ml final conc.). Cells were incubated with 200 μl of DMEM, FBS (10%) containing 2 μl NucBlue™ Live ReadyProbes™ Reagent Hoechst 33342 (ThermoFisher Scientific) and *O*-acetylated benzylguanine fluorescein isothiocyanate (ac-BG-FITC, 2 μM final conc.). After fixation with formaldehyde (3.7% final conc.) and permeabilization with Triton X-100 (Carl Roth, #3051.3, 0.1% final conc.), cover slips were mounted on microscope slides using Dako Fluorescence Mounting Medium (AgilentDako) and dried over-night at 4°C. Images were taken with an AXIO Observer.Z1 (Zeiss), Colibri.2 light source under 63x magnification.

### Functional CTNNB1 assay

Editase expressing cells were transfected as described above in technical duplicates (SNAP-CDAR-S) or quadruplicates (Cas RESCUE-S). Cells were transfected with either M50 Super 8x TOPFlash (Addgene, #12456) or M51 Super 8x FOPFlash (Addgene, #12457) and with pcDNA3.1 expressing Renilla Luciferase for normalization. Samples were also transfected either empty, or with CTNNB1 T41-targeting (snap)_2_-gRNA or gRNA expressing plasmid (RESCUE-S), or with PPIB R7C-targeting (snap)_2_-gRNA or gRNA expressing plasmid (RESCUE-S), or with CTNNB1 T41-targeting NH_2_-gRNA or plasmid expressing no gRNA (RESCUE-S) in a 96-well format. Cells were lysed with 30 μl (SNAP-CDAR-S) or 20 μl (Cas RESCUE-S) per well of Passive Lysis Buffer (Promega) and shaken for 15 min. at room temperature. Luciferase signal was measured as described before (26) using the Dual-Luciferase® Reporter Assay System (Promega) following manufacturer's instructions with a Spark 10 M plate reader (Tecan). Briefly, 10 μl of each replicate (two of the four wells for RESCUE-S were pooled) were measured by addition of 35 μl of Luciferase Assay Reagent II (LAR II, Promega) and 35 μl of Stop & Glo® Reagent, and measured for 10 seconds, respectively. Editing yield was determined as described above. All luminescence measurements and editing yield determinations were conducted in biological triplicates.

### Editing of STAT3 pS727

3 × 10^5^ Flp-In T-Rex cells expressing SNAP-CDAR-S were seeded in a 24-well format in DMEM, FBS (10%), D (10 ng/ml) to induce transgene expression. After 24 h, 3.2 × 10^5^ cells were transfected with 20 pmol of STAT3 S727F-targeting NH_2_-gRNA, (snap)_2_-gRNA, or PPIB R7C-targeting (snap)_2_-gRNA (quadruple of 96-well format) using 2 μl Lipofectamine™ RNAiMAX (invitrogen) per transfection. This transfection was repeated on day 2, 4 and 6 after the first transfection. Endpoint was at day 8. A fraction of cells was used for RNA isolation and editing analysis as described above. The rest of the cells was lysed with RIPA lysis and extraction buffer (ThermoFisher Scientific) supplemented with cOmplete™ Tablets, Mini, EDTA-free *EASY*pack Protease Inhibitor Cocktail (Roche) and PhosStop *EASY*pack (Roche). Protein concentration was determined by Pierce™ BCA Protein Assay Kit (ThermFisher Scientific) in a Tecan Plate Reader. 30 μg of total protein was run on a Novex™ WedgeWell™ 8 to 16%, Tris-glycine, 1.0 mm, Mini Protein Gel (ThermoFisher Scientific) with 200 V for 60 min. Blotting was performed with a Mini Trans-Blot Cell^®^ (BioRad) at 100 V for 60 min. For protein detection, membranes were incubated with monoclonal anti-β-actin antibody produced in mouse (Sigma, 1:5000 dil.) and either Stat3 (DRZ2G) Rabbit mAb (CellSignaling, 1:1000 dil.) or P-Stat3 (S727) (D8C2Z) Rabbit mAb (CellSignaling, 1:1000 dil.). Membranes were then incubated with peroxidase AffiniPure goat anti-mouse IgG (Jackson Immuno Research, #115-035-003, 1:10000 dil.), and peroxidase AffiniPure goat anti-rabbit IgG (Jackson Immuno Research, #111-035-003, 1:10000 dil.). Images were taken with Odyssey FC Imager (Li-Cor^®^ Biosciences).

### Next generation sequencing

Cells expressing SNAP-CDAR-S or Cas RESCUE-S were transfected with or without (snap)_2_-gRNA (2.5 pmol) or gRNA expression vector (300 ng), respectively, targeting PPIB R7C in technical duplicates under constant Dox (10 ng/ml) induction. Total RNA was isolated with RNeasy MinElute Cleanup Kit (Qiagen) following manufacturer's instructions. After DNase I (NewEngland BioLabs) digest, samples were again purified with RNeasy MinElute Cleanup Kit. Editing yields were first determined by Sanger Sequencing, as described above. Next Generation Sequencing was performed by CeGaT with a NovaSeq6000 (Illumina) to generate 50 Mio. 2 × 100 bp paired-end reads per sample. Library was prepared with TruSeq Stranded mRNA Kit (Illumina). Lanes of raw RNA sequencing data of same samples were pulled together, and adapter sequences were trimmed with Trim Galore (v0.6.5; http://www.bioinformatics.babraham.ac.uk/projects/trim_galore/). Sequencing alignment to the human reference genome (hg19) was performed using STAR (v. 2.7.10a). hg19 and the RefSeq annotation are publicly available at the genome browser at UCSC. For alignment uniquely mapped (STAR option: –outFilterMultimapNmax 1) reads were considered to prevent multimapping of regions of high similarity. Next, data (bam files) were deduplicated, sorted, and indexed using SAMtools (v1.9; http://samtools.sourceforge.net). SNVs calling was performed with REDItools (v2; https://github.com/tflati/reditools2.0). Developers’ recommendations were taken into consideration for preceding data preparation. As previously performed, only high-quality sites (min. MeanQ > 30 in REDItools2) were considered. Editing site were called when well-covered i.e. minimum 50 reads (in summary of duplicates per sample), showing ≥5% editing frequency compared to the control. For sites matching criteria, fisher's exact test was performed. Significance was defined for all samples showing an adjusted *P*-value < 0.01. Genomic coordinates were annotated with Variant Effect Predictor (VEP; v102) using the following command line:

vep –input file input..txt –fasta reference.fa –output_file output.txt –species homo_sapiens –tab –cache –dir_cache ../Human/dir –no_check_variants_order –transcript_version –canonical –ccds –hgvs –symbol –gene_phenotype –pubmed –variant_class –pick –offline –force_overwrite

### Further information

For more detailed protocols and guide RNA sequences, please see additional Supplementary Information and additional Supplementary files. Detailed information on reagents, enzymes, antibodies, and kits as well as cell lines used in this study are presented in the Supporting Information.

## RESULTS

### The APOBEC1-SNAP tool suffers from low C-to-U editing yields and limited programmability

Recently, we demonstrated the harnessing of the murine APOBEC1 deaminase for site-directed C-to-U RNA base editing. For this, the SNAP-tag was fused to the C-terminus of the full length APOBEC1 enzyme, resulting in an editor called APO1S (Figure 1A) (20). In transgenic cell lines that co-express APO1S together with SNAP-ADAR1Q, moderate editing yields were achieved on an eGFP reporter gene, but editing yields on the endogenous GAPDH transcript stayed below 20%. By targeting the eGFP reporter, we now tried several means to improve editing yields. On the guide RNA side, the positioning of the guide RNA four to five nucleotides upstream with respect to the target cytidine was most important (Figure 1B, C). On the protein side, the localization of the editing enzyme to the cytosol was particularly necessary (Figure 1D, E, Supplementary Figures S1-S6). Nevertheless, the APO1S editor suffered overall from low editing yields on endogenous targets and from low programmability (Figure 1F), meaning that transfer to endogenous transcript was particularly difficult followed by notable guide RNA-dependent bystander editing when on-target editing was successful (Supplementary Figure S7).

### The SNAP-CDAR-S tool combines high editing yields with excellent programmability

**Figure 2.**
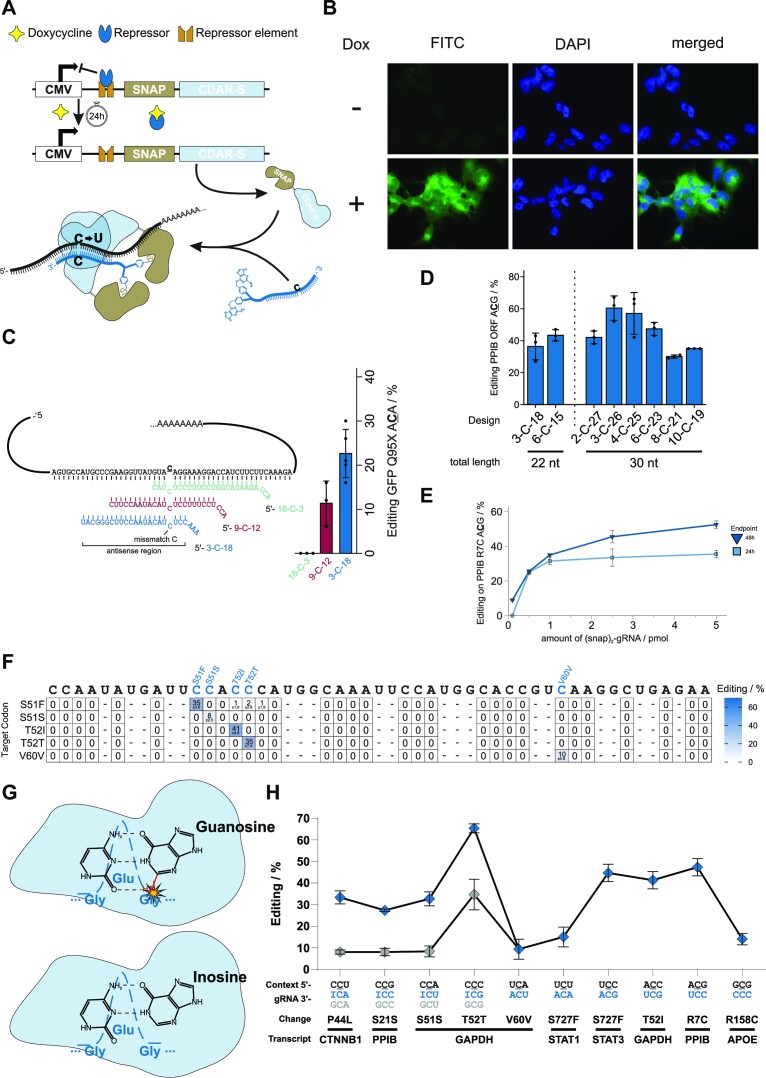
Guide RNA design and performance of the SNAP-CDAR-S tool. (**A**) Scheme of the doxycycline-inducible SNAP-CDAR-S tool and the guide RNA-dependent editing reaction. (**B**) Analysis of SNAP-CDAR-S transgene expression and localization by fluorescence microscopy. SNAP-CDAR-S was stained with SNAP-tag-reactive BG-FITC (green channel). (**C**) Dependency of editing yield of the Q95X site (5′-ACA) in the eGFP reporter gene on the positioning of the guide RNA (all 22 nucleotides length, 2′OMe modification on all nucleotides except for mismatch C and flanking nucleotides) relative to the target site (symmetric versus asymmetric). (**D**) Fine-tuning of guide RNA length (22, 30 nt) and positioning (of mismatch cytidine C) for optimal editing performance. (**E**) Dependency of RNA editing yield on the amount of transfected guide RNA (pmol/96 well). (**F**) Programmability and precision of the SNAP-CDAR-S tool. Five guide RNAs (each 6-C-23, 2′OMe gapmer design) against five nearby cytidine sites on the endogenous GAPDH transcript, each with a distinct codon context, were applied and the on-target and the C-to-U and A-to-I bystander off-target editing was determined by Sanger sequencing. (**G**) Scheme explaining the positive effect of inosine in guide RNAs for targeting 5′-CCN codons. Pairing of the 5′ cytidine in a 5′-CCN context with a guanosine leads to a steric clash of CDAR′s glycine with the guanosine's exocyclic NH_2_-group (28). Inosine lacks that NH_2_-group thus avoiding steric clash. (**H**) Codon scope of the SNAP-CDAR-S tool and effect of inosine in 5′-CCN codons. Given are editing yields for various codons on various endogenous transcripts when applying non-optimized guide RNAs of the standard design (6-C-23, 2′OMe gapmer). For each of the four 5′-CCN codons (*N* = U, C, A, G), the editing yields of guide RNAs are compared that contained either an inosine (I) or a guanosine (G) opposite the cytidine preceding the on-target cytidine. Data in (C), (D), (E), (F) and (G) are shown as the mean ± s.d. of *N* ≥ 2 independent experiments.

In contrast, the Cas-13-mediated C-to-U editing tool called RESCUE applies a C-to-U deaminase that was evolved from the A-to-I deaminase ADAR2 (24), and shares with ADAR2 its strong substrate preference for double-stranded RNA. Indeed, the RESCUE tool seems to have much better programmability and on-target editing was reliably obtained when the target site was positioned inside the guide RNA / mRNA duplex. However, Cas13-mediated C-to-U editing suffers from global and local C-to-U and A-to-I off-target editing, and attempts to create more precise tools, like Cas13-RESCUE-S, came along with largely reduced on target editing yields, hardly above 10% on endogenous transcripts (24). However, we were wondering how the engineered cytidine deaminase acting on RNA (CDAR) domain would work in the context of a SNAP-tagged fusion protein (12). For this, we fused the evolved, high-fidelity deaminase of the RESCUE-S tool to the C-terminus of a SNAP-tag (11,14) to obtain the SNAP-CDAR-S tool (Figure 2A). We stably integrated a single copy of the SNAP-CDAR-S transgene into HEK 293 cells by using the Flp-In approach, (14,19) and found homogenous transgene expression under control of doxycycline. Similar to the closely related A-to-I editing enzyme SNAP-ADAR2Q (Supplementary Figure S1), the SNAP-CDAR-S tool was mainly localized to the cytosol (Figure 2B).

Our initial guide RNA design was inspired from our experience with the SNAP-ADAR tool and was tested for the editing of a 5′-ACA codon in a co-transfected eGFP reporter transcript. Initial guide RNAs were 22 nt long, chemically modified by 2′-O-methylation outside the target base triplet (27), which is the targeted cytidine plus its two closest neighboring bases, and carried a (snap)_2_ self-labeling moiety (20) at the 5′-end for the recruitment of two SNAP-CDAR-S effectors per guide RNA. The exact composition, sequence and chemistry, of all guide RNAs can be found in the Supplementary Information (Supplementary Table T1a). While the SNAP-ADAR tool prefers a relatively central positioning of the target nucleobase, the SNAP-CDAR-S effector gave clearly better yields when the target cytidine was located near the 5′-terminus of the guide RNA, e.g. design 3-C-18 in Figure 2C, in good agreement with data from the Cas13-RESCUE (24) tool. Next, we took a closer look at the guide RNA design for the editing of the endogenous PPIB transcript, specifically, by targeting a 5′-ACG codon in its coding region (ORF). Here, we varied the length of the guide RNA (22 nt and 30 nt) and the positioning of the guide RNA relative to the target cytidine, see Figure 2D. With 60% editing yield, we found the best performing guide RNA to be 30 nt long, positioning the targeted cytidine close to the 5′-end (position 4, 3-C-26) of the guide RNA in the substrate duplex. However, also other guide RNA designs gave good editing yields and the optimal design might vary to some extent for each target sequence, as suggested for the original Cas13-RESCUE-S tool (24). As we sought to determine a universal guideRNA design, we compared editing yields on further endogenous transcripts putting the target C in position 4 (3-C-26) or position 7 (6-C-23, Supplementary Figure S8). In contrast to the target site on PPIB, positioning target C at position 7 showed substantially higher editing yields for all selected sites (Supplementary Figure S8). In addition, we transferred designs previously reported (24) ideal for four endogenous sites to our SNAP-CDAR-S tool and compared them to our 6-C-23 standard designs (Supplementary Figure S9). For all targets, our standard design gave similar or even better editing yield then the previously described ideal design. This indicated that 6-C-23 can be considered a universal design for the SNAP-CDAR-S tool. We then continued to analyze the performance further. Editing yields were saturating when ≥2.5 pmol/96 well (20 nM) guide RNA were transfected (Figure 2E). To characterize the scope, programmability and precision of the SNAP-CDAR-S tool, we targeted five different guide RNAs (all 30 nt, 6-C-23, and 2′-OMe) against five different cytidine bases, which were all located in close proximity in the ORF of the endogenous GAPDH transcript and determined on-target as well as C-to-U and A-to-I bystander editing yields. We found excellent programmability, with good on-target yields (8% to 41%) and with lacking bystander editing (detection limit Sanger sequencing ca. 5%) at neighboring cytidine or adenosine bases (Figure 2F, the same was found for an alternative 3-C-18 guide RNA design, see Supplementary Figure S10). 2′-O-Methylation was shown in the past to block bystander A-to-I editing very efficiently in SNAP-ADAR tools (14,27), and this may contribute here to the high precision of the targeted editing too. However, we were not fully satisfied with the editing yield at the 5′-CCA codon (GAPDH S51S), which achieved only 8% with the best design (6-C-23, a 3-C-18 design gave < 5%). This limited scope was also reported for the Cas13-based RESCUE tool (24) and resembles the codon preference of the ancestor ADAR2 (25) protein. A recent structural analysis of the ADAR2 deaminase bound to a dsRNA substrate revealed a steric clash between the peptide backbone of glycine 489 and the minor groove face of G = C base pairs residing at the 5′ neighboring position to the target adenosine (28). This steric clash could be relaxed by replacing the 5′-neighboring G = C base pair with sterically less demanding I = C base pair (lacking an exocyclic amino group), simply by pairing the 5′-CCA target codon with a 5′-UCI sequence in the guide RNA (Figure 2G). Indeed, we found a 3-fold improved editing yield of 32% for the respective site in GAPDH (Figure 2H, Supplementary Figure S10B). We then systematically tested the principle for all four potential 5′-CCN codons (N = A, U, G, C) and found that an inosine base opposite the 5′-neighboring cytidine always improved editing at the targeted cytidine base (Figure 2H). Even for the well-edited 5′-CCC codon (34%), we could still achieve a notable gain in editing yield (66%).

### SNAP-CDAR-S clearly outperforms Cas13-RESCUE-S on endogenous targets

**Figure 3.**
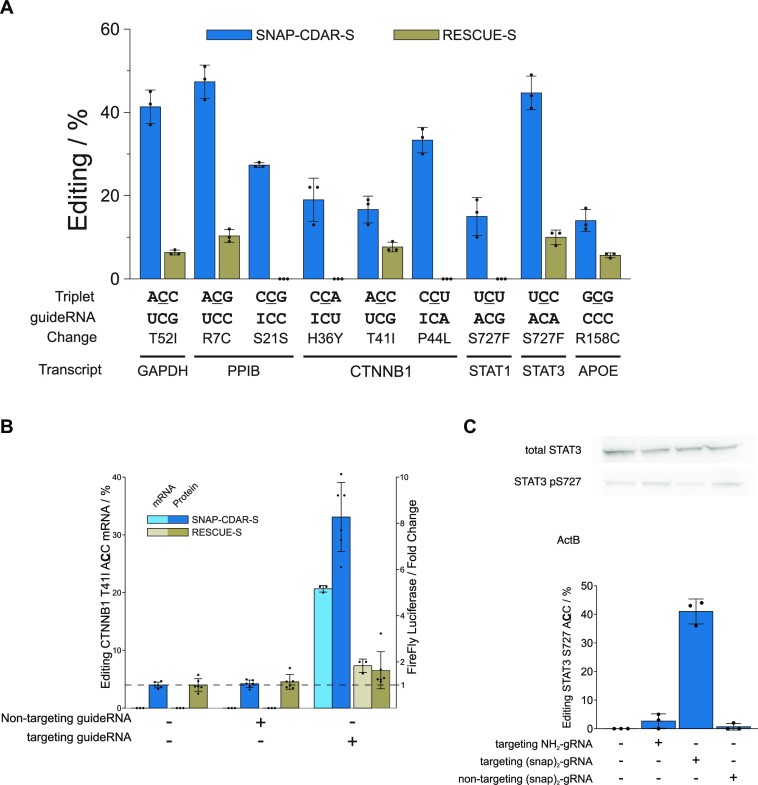
Benchmark with Cas13-RESCUE-S on endogenous targets and applications. (**A**) Comparison of editing yields at various sites on various endogenous targets and one disease-relevant cDNA (APOE) comparing SNAP-CDAR-S with standard guide RNAs (30 nt, 6-C-23, 2′OMe gapmer) versus Cas13 RESCUE-S with plasmid-borne optimized Cas guide RNAs. Both editing enzymes were expressed from the same single genomic locus. (**B**) Comparing both tools, SNAP-CADR-S versus Cas RESCUE-S, for the activation of β-catenin by RNA editing. Given are C-to-U editing yields (T41I) and the luminescence-based read-out of pathway activation. For further controls, see Supplementary Figure S11. (**C**) Editing of the regulatory phospho-site serine 727-to-glycine in STAT3, read-out of editing yield by Sanger sequencing, and amount of total STAT3 and pS727 STAT3 protein by western blot. Data in (A), (B) and (C) are shown as the mean ± s.d. of *N* = 3 independent experiments.

To benchmark the SNAP-tagged tool with the Cas13-based tool, we generated an analogous 293 Flp-In T-REx cell line stably expressing the Cas13-based RESCUE-S on doxycycline induction. In the original work (24), RESCUE-S has always been applied by means of transient overexpression, however, this often leads to high variability in editing yields and artifacts in off-target analyses (12). We tested both editing tools side-by-side for the editing of eight different sites on five different endogenous transcripts (GAPDH, PPIB, CTNNB1, STAT3, STAT1) and one disease-relevant cDNA (APOE). Most target sites were taken from the original Cas13-RESCUE-S publication (24) so that optimal Cas guide RNAs have already been reported for each of them (Supplementary Table T1b). We repeated these experiments by transfecting 300 ng/96 well of the plasmid-borne optimal guide RNAs into the stable Cas13-RESCUE-S cells. The guide RNAs for the SNAP-CDAR-S cell lines were not optimized, but we simply transfected 5 pmol/96 well chemically stabilized, 30 nt guide RNAs of the 6-C-23 standard design. Nevertheless, the SNAP-CDAR-S tool clearly outcompeted the Cas13-RESCUE-S tool on all eight targets, achieving editing yields between 10% and 50% (Figure 3A), while the editing yields of the Cas13-RESCUE-S tool did not achieve editing yields above 10%, in accordance with the original report (24). For four targets, only SNAP-CDAR-S, but not Cas13-RESCUE-S, was able to achieve detectable editing (PPIB S21G, CTNNB1 H63Y and P44L, STAT1 S727F). Interestingly, three out of these four examples target 5′-CCN codons, which is readily done by the SNAP-CDAR-S approach with inosine-containing guide RNAs, highlighting that the SNAP-CDAR-S approach does not only give generally higher editing yields but also increases the codon scope towards 5′-CCN sites.

Several of the indicated targets are of clinical interest. The removal of threonine 41 from the β-catenin protein inactivates a degron and thus stabilizes the protein. (29) Enhanced levels of β-catenin could be applied to boost liver regeneration or wound healing transiently (30). We benchmarked the activation of the Wnt pathway by the SNAP-CDAR-S versus its analog Cas13 tool in a plasmid-borne luciferase assay, following a protocol reported before (24). While the SNAP-tagged tool achieved 21% editing yield and an 8-fold increase in β-catenin activity (Figure 3B, Supplementary Figure S11), the Cas13-RESCUE-S tool gave only 7% editing yield and 1.6-fold increase. The STAT3 protein (signal transducer and activator of transcription 3) is a multifunctional signaling molecule, which acts as a transcription factor in the nucleus, or translocates to the mitochondrium, and modulates immune response and metabolism. Its hyperactivation plays an important role in autoimmune disease, sterile inflammation, and cancer (31). Here, we removed serine 727 from STAT3, a functionally important phosporylation site. We could achieve up to 41% serine-to-glycine editing, which was accompanied by a visible reduction in S727 phosphorylation as determined by Western blot (Figure 3C, Supplementary Figure S12). Finally, we aimed at introducing a protective genotype into the apolipoprotein E (APOE) transcript, introducing the rs7412 SNP (R158C), which could transfer the neutral ϵ3 allel (ca. 78% caucasian carriers) into the protective ϵ2 allel, which was shown to largely reduce the risk for atherosclerosis (32). However, given the non-preferred nature of the codon (5′-GCG), and the very high GC content of the surrounding sequence space, an editing yield of only 14% was achieved, still clearly outcompeting the Cas13 tool (Figure 3A).

### Both tools show moderate global A-to-I and C-to-U off-target editing

**Figure 4.**
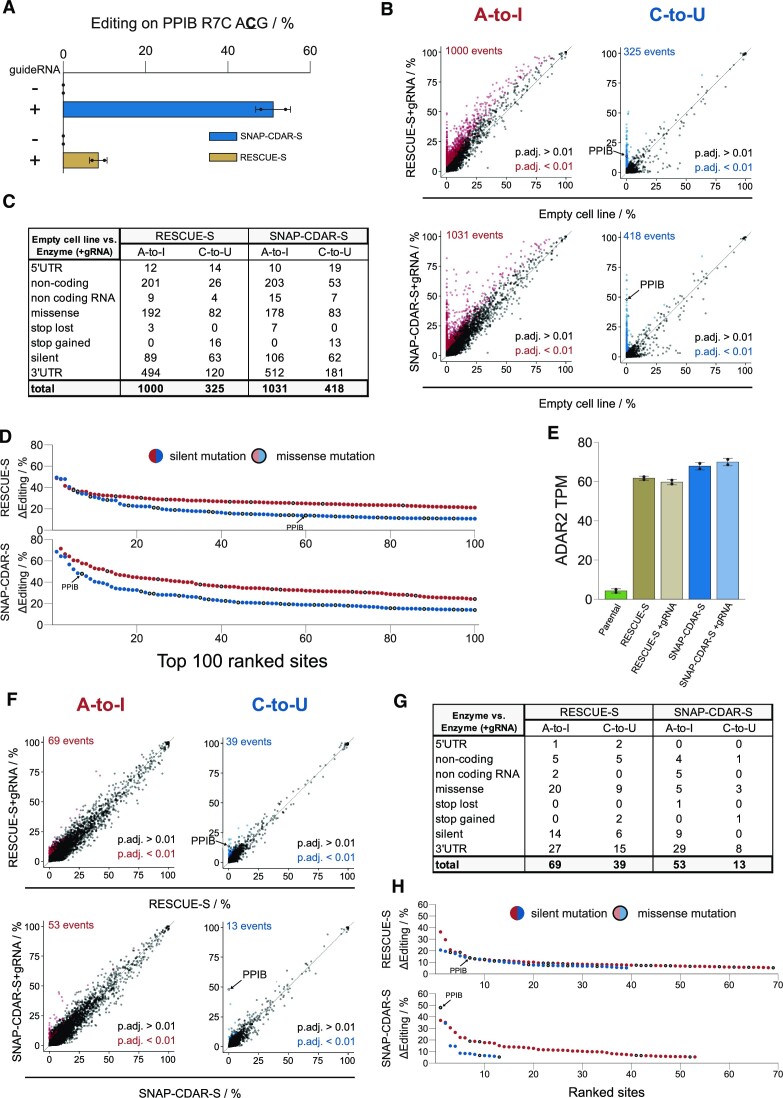
Precision of SNAP-CDAR-S versus Cas13-RESCUE-S as determined by NGS. (**A**) Editing yields as determined by Sanger sequencing prior to NGS. (**B**) Plot of total off-target events of the respective effector + guide RNA against the empty cell line. Significantly differently edited sites (adjusted *P*-value < 0.01) are colored in red (A-to-I) and blue (C-to-U), respectively. (**C**) Total off-target effects sorted by categories. (**D**) Changes in editing yields (ΔEditing) of the 100 top-ranked editing sites color-coded for A-to-I (red) and C-to-U (blue) editing. The on-target editing event is marked by an arrow. Missense and silent mutations are indicated by empty and filled symbols, respectively. (**E**) Transgene expression levels as determined by TPM values of ADAR2 (ADARB1). Given that CDAR-S is an ADAR2 deaminase mutant, both transgenes (SNAP-CDAR-S, Cas13-RESCUE-S) were annotated as such. ADAR2 itself is not expressed in the parental 293 cell line. Both transgenes were expressed to comparable TPM levels and did not change upon presence of the guide RNA. (**F**) Plot of guide RNA-dependent off-target events of the respective effector + guide RNA against the respective effector. Significantly differently edited sites (adjusted *P*-value < 0.01) are colored in red (A-to-I) and blue (C-to-U), respectively. (**G**) Guide RNA-dependent off-target effects sorted by categories. (**H**) Changes in editing yields (ΔEditing) of the top-ranked editing sites, color-coded for A-to-I (red) and C-to-U (blue) editing. The on-target editing event is marked by an arrow. Missense and silent mutations are indicated by empty and filled symbols, respectively. Significance in panels (B) and (F) was tested by Fisher's exact test (two-sided), *n* = 2 independent experiments.

We used next-generation sequencing of the poly(A)+ transcriptome (10 GB per condition) to assess the transcriptome-wide A-to-I and C-to-U off-target editing of the SNAP-CDAR-S versus its analog Cas13-RESCUE-S. We took RNA from cells expressing the respective editing effector in the presence and absence of the respective guide RNA and compared them to Flp-In T-REx cells not expressing an engineered effector (14). First, we compared the editing reactions against the empty Flp-In T-REx cell line and were able to detect the on-target editing event (PPIB Arg7Cys) with editing yields of 48% for SNAP-CDAR-S and 14% for Cas13-RESCUE-S, which were confirmed by Sanger sequencing (Figure 4A). Beside the on-target editing, we found around 1000 A-to-I and three to four hundred C-to-U off-target events for both effectors (Figure 4B). As seen for ADAR-based effectors before, (10,12,33) A-to-I off-target editing was a combination of enhanced editing at known sites and editing at novel sites, whereas the large majority of C-to-U off-target editing were novel sites. We further analyzed the outcomes of the editing reactions and found that only a moderate number of all editing events resulted in missense mutations (Figure 4C). At less than ten missense sites, the change in the off-target editing yield was increased above 25%, indicating that most missense sites are only marginally affected (Figure 4D). The patterns between the two effectors were very similar, which was expected given that the CDAR domain of both editing tools is identical. The presence of the editing tools gave no larger changes in gene expression (Supplementary Figure S13), and both editing effectors were expressed to similar TPM levels (Figure 4e). Finally, we analyzed the guide RNA-dependent changes in editing. Clearly, the vast majority of off-target editing came from the presence of the editing enzymes and was guide RNA-independent (Figure 4F, G). The guide RNA-dependent C-to-U editing was very clean for the SNAP-CDAR-S tool. In contrast, the on-target editing with Cas13-RESCUE-S was covered by a small number of further editing events. This became also visible when we plotted all guide RNA-dependent changes in editing yields (Figure 4H). While the on-target site gave the largest ΔEditing value for the SNAP-CDAR-S tool, the Cas13-RESCUE-S tool gave six A-to-I and another six C-to-U off-target events with higher change in editing level. Overall, both enzymes were expressed to comparable TPM levels, gave very similar patterns and levels of global off-target editing and mainly differed in the 5-fold higher on-target editing yield of the SNAP-CDAR-S tool.

## DISCUSSION

In comparison to the APOBEC1 enzyme, the CDAR domain, evolved from ADAR2, performs considerably better in targeted RNA base editing tools. While the CDAR domain was taken from the Cas13-mediated RESCUE approach (24), we could show here that this deaminase domain works particularly well when the self-labeling SNAP-tag is applied as the RNA-targeting mechanism. Compared to the Cas13-RESCUE-S, the SNAP-CDAR-S gave reliably higher on-target yields with less bystander editing, while the global A-to-I and C-to-U off-target effects were comparable. A reason for the superior efficiency of the SNAP-tagged tool might be the chemical design and the covalent bond that tethers the guide RNA to the SNAP-tag and may foster the encounter of guide RNA, target RNA and editing effector (12,14). Regarding the design of the guide RNAs with respect to chemical modifications, we found that lessons learned from the engineering of the closely related SNAP-ADAR tool (14,27) could be largely transferred. In particular, the general guide RNA design with 2′-O-methylation at the ribose moieties outside the base triplet and the usage of non-encodable bases like inosine opposite 5′-CCN codons have contributed to the improved performance so that editing yields between 10% and 50% are regularly achieved in 5′-HCN (H = C, A, U) codons, and only 5′-GCN codons remain challenging. To our knowledge, our data is the first report of stable integration of the Cas13-RESCUE-S tool and shows that it functions as well under genomic integration as it does via plasmid overexpression. Even though Cas13-RESCUE-S was presented as a high-fidelity enzyme with largely reduced global A-to-I off-target editing before (24), there still remains notable A-to-I as well as C-to-U off-target editing, which might have been underestimated in the prior study, where off-target analysis was done on reporter cDNA under co-transfection of the editing enzyme. Our work further allows to compare the A-to-I off-target effects of the SNAP-CDAR-S deaminase directly to the related, stably integrated wildtype and hyperactive (E488Q) mutant of SNAP-ADAR2 (14). While the off-target A-to-I editing of the SNAP-CDAR-S tool is clearly below that of the hyperactive, off-target-prone SNAP-ADAR2 E488Q mutant (14), the SNAP-CDAR-S tool still has notably frequent off-target A-to-I editing when compared to the wildtype SNAP-ADAR2 enzyme (see Supplementary Figure S14), which is clearly not yet optimal for a C-to-U editing enzyme and may require further engineering efforts to generate a pure C-to-U editing enzyme. Recently, improved C-to-U editing tools based on the Cas13-RESCUE platform have been described (34,35). Engineering efforts allowed to largely reduce the size of the Cas protein, e.g. to enable AAV-mediated delivery. However, these tools remain built on the original C-to-U deaminase domain taken from the RESCUE(-S) tool so that global C-to-U and in particular A-to-I off-target editing remains observable. In contrast, a split version of the CDAR domain has recently been described to strongly improve editing precision, e.g. by largely abolishing such global off-target editing. (36) Specifically, the tool uses an orthogonal trans-tethering approach, steering one CDAR half via an MS2/MCP and the other half via a λN/BoxB interaction to the target mRNA. While this was working in principle, the C-to-U editing efficiency remained very low (e.g. around 5%). Finally, C-to-U editing tools have been constructed based on the RNA/DNA editing enzyme APOBEC3A, including the Cas13-based tool CURE (22) and the PUF domain-based tool REWIRE (37). While both tools enable programmable editing, they also come with specific limitations. These include global off-target C-to-U editing at the RNA and DNA level, but also a strongly limited codon scope, e.g. 5′-UCR (R = A, G). Overall, the SNAP-CDAR-S tool adds a reliable and efficient enzyme with large codon scope to the tool box for targeted RNA base editing. It may also be worth mentioning that the SNAP-CDAR-S tool avoids protein parts taken from bacterial origin, like Cas proteins, which raise concerns regarding immunogenicity upon their perpetual expression, (38) which would be required in many therapeutic settings. Even though editing efficiency and precision of SNAP-CDAR-S are not yet perfect, the tool clearly outperforms the original Cas13-based RESCUE-S and lays a basis for further engineering in the future.

## Supplementary Material

gkad598_Supplemental_Files

## Data Availability

The raw NGS data is uploaded on the NCBI BioProject Server under ID PRJNA948645.
