# Supplementary material for: Precise and efficient C-to-U RNA base editing with SNAP-CDAR-S
Source: Nucleic Acids Res. 2023 Jul 18;51(15):e84. doi: 10.1093/nar/gkad598 (PMC10450179; doi:10.1093/nar/gkad598)
Supplement: gkad598_Supplemental_Files [file gkad598_supplemental_files.zip › revised Supporting Information_clean.pdf]

Precise and efficient C-to-U RNA Base Editing with SNAP-CDAR-S

Ngadhnjim Latifi[1], Aline Maria Mack[1], Irem Tellioglu[2,3], Salvatore Di Giorgio[2] and Thorsten Stafforst[1,4*]

[1]Interfaculty Institute of Biochemistry, University of Tübingen, Auf der Morgenstelle 15, 72076 Tübingen, Germany
[2]Division of Immune Diversity (D150), German Cancer Research Center (DKFZ), 69120 Heidelberg, Germany.
[3]Faculty of Engineering, University of Heidelberg, 69120 Heidelberg, Germany
[*]correspondence to thorsten.stafforst@uni-tuebingen.de
[4]Gene and RNA Therapy Center (GRTC), Faculty of Medicine University Tuebingen

Table of contents

Cells stably expressing editases **1**

Generation of stable cell lines *1*

Cloning of constructs 1

Microscopy *2*

Editing with Snap-editases **3**

snap/(snap)$_2$-gRNA synthesis *3*

PAGE *3*

Transfection of Snap-editases expressing cells *4*

Editing with RESCUE-S **4**

Cloning of guideRNA oligos *4*

Transfection of RESCUE-S cell lines *5*

Analysis of editing yield *6*

Editing analysis of APOE **6**

CTNNB1 Assay **7**

Transfection conditions *7*

FireFly Luciferase expression and editing yield *8*

STAT3 Assay **8**

Next Generation Sequencing **10**

Supplementary Data **12**

Cells stably expressing editases

Generation of stable cell lines

For creation of cell lines stably expressing editases, the Flp-In™ T-REx™ system (Invitrogen, #R78007) was used. Hek 293 Flp-In™ T-Rex™ cells were cultivated in Dulbecco's modified eagle medium (DMEM; Life Technologies, #41965062) with fetal bovine serum (FBS; Life Technologies, #10270, 10% final conc.), Zeocin™ Selection Reagent (Z; Invitrogen, #R25001, 100 µg/ml final conc.), and Blasticidin S Hydrochloride (B; Blasticidin S Hydrochloride, Carl Roth, #CP14.2, 15 µg/ml final conc.) For generation of cell lines, 1.6 x 10^6 cells were seeded in a 6 cm dish in DMEM +10% FBS, +Z, +B. After 24 h medium was switched to DMEM + 10% FBS without antibiotics 1 h prior to transfection. Cells were transfected with 4 µg of plasmid in a 1:9 ratio of pOG44 Flp-Recombinase expression vector (invitrogen, #V600520) and pcDNA™5/FRT Mammalian Expression Vector (Invitrogen, #V601020) the corresponding editase with 12 µl Lipofectamine™ 2000 (ThermoFisher Scientific, #11668019) in 600µl Opti-minimal essential medium I (Opti-MEM™ I; Life Technologies, #11058021) final volume. After 24 h, medium was changed to selection medium swapping Zeocin with CELLPURE® Hygromycin B solution (H; Carl Roth, #CP12.1, 100 µg/ml final conc.) After 2 weeks of selection, cells were taken into culture in DMEM +10% FBS, +B, +H.

Cloning of constructs

All construct for generation of cell lines stably expressing them were cloned into the pcDNA™5/FRT Mammalian Expression Vector (Invitrogen, #V601020).

Apo1S constructs

We added localization tags to Apo1S at the C-terminus. For this, tags were ordered as synthetic oligonucleotides, which were annealed and ligated into the pcDNA™5. Oligo pairs (1 µl of either oligo 100 µM in H2O) were diluted in 200 µl of water. 39 µl of dilution were

SNAP-CDAR-S constructs

Mutations for transversion of ADARs adenine deamination activity to cytidine deamination (Abudayyeh, O. O., et al., Science 365.6451 (2019): 382-86) were transferred to the deaminase domain of our SNAP-ADAR2Q construct and synthesized by ThermoFisher Scientific GeneArt Services.

Microscopy

Glass cover slips were inserted in 24-well plate well and coated with Poly-(D)-lysine hydrobromide (Sigma-Aldrich, #P6407-5MG, 0.1 mg/ml final conc. in H_2O) for 30 min. After subsequent washing with H2O and Phosphate-buffered saline (PBS; 137 mM NaCl, 2.7 mM KCl, 10 mM Na_2HPO_4, 2 mM KH_2PO_4), the slips were dried for 30 min. under UV irradiation. After 30 min. more of drying, the cover slips were ready for use. 5 x 10^4 Flp-In™ T-Rex™ cells expressing SNAP-editases were seeded on the cover slips in DMEM, FBS (10%), B, H and Doxycycline hyclate BioChemica (D; PanReacAppliChem, #24390-14-5, 10 ng/ml final conc.) to induce expression. After 24 h, cells were washed with PBS and incubated for 30 min. with staining solution. Staining solution consisted of DMEM, FBS (10 %) containing 2 µl NucBlue™ Live ReadyProbes™ Reagent Hoechst33342 (ThermoFisher Scientific, #R37605) and acetylated benzylguanine fluorescein isothiocyanat (ac-BG-FITC, 2 µM final conc.) in 200 µl final volume. Cells were fixated by addition of 21.6 µl of 37 % of aqueous formaldehyde (3.7 % final conc.) and incubation for 3 min. Cells were then washed three times with PBS (3 x 200 µl) and permeabilized with 200 µl PBS containing Triton X-100 (Carl Roth, #3051.3, 0.1 % final conc.) by incubation for 15 min. For microscopy, cover slips were mounted on microscope slides using Dako Fluorescence Mounting Medium (AgilentDako, #S302380-2) and dried over-night at 4 °C. Images were taken with an AXIO Observer.Z1 (Zeiss), Colibri.2 light source under 63x magnification.

Editing with Snap-editases

snap/(snap)$_2$-gRNA synthesis

gRNAs were designed and purchased by Eurogentec or SigmaAldrich with a 5’-C6-Amino linker or a 3’-C7-Amino linker referred to as NH_2-gRNA. Pre-activation of snap-linker was carried out with EDCI. 120 nmol of snap-linker (2 µl of 60 mM in DMSO) were incubated with 224 nmol of EDCI (2 µl of 112 mM in DMSO), 306 nmol of NHS (2 µl of 153 mM in DMSO), and 2 µl of DIPEA (5% solution in DMSO) for 60 min at 30°C. After pre-activation, 4 µl of DIPEA (5% solution in DMSO) were added to 8.5 µl of NH2-gRNA solution (50 µg of 6 µg/µl in H2O). For conjugation, half of the pre-activation mix was added to the NH2-gRNA and incubated for 30 min at 30°C. The rest of the pre-activation mix was added and incubated for 60 min at 30°C.

Pre-activation of snap2-linker was carried out DIC. 120 nmol of snap2-linker (2 µl of 60 mM in DMSO) were incubated with 540 nmol of DIC (2 µl of 270 mM in DMSO), 920 nmol of NHS (2 µl of 460 mM in DMSO), and 2 µl of DIPEA (5% solution in DMSO) for 4 h at 45 °C or over-night at 37 °C. Mix was lyophilized to remove residual DIC and subsequently dissolved in 12 µl of DIPEA (1.7 % in DMSO). 8.5 µl of NH_2-gRNA solution (50 µg of 6 µg/µl in H_2O) was added to the pre-activated linker and incubated for 2 h at 37 °C.

PAGE

Successful coupling was determined by separation on a 20% 5M urea PAGE. Gels were cast in large glass plates separated by 0.8 mm spacers. Mix for gel was made with 204 ml of ROTHIPHORESE®Sequencing gel concentrate (25 %, Carl Roth, #3043.1), 13 ml of ROTHIPHORESE®Sequencing gel buffer concentrate (50 %, Carl Roth, #3050.1), 13 ml of H_2O, 650 µl of Ammonium peroxydisulphate (Carl Roth, #9592.3, 10% solution in H_20), and 65 µl of tetramethylehtylenediamine (TEMED; Carl Roth, #2367.3). Cast gels were allowed to solidify at room temperature over-night. Each sample was supplemented with 5 µl of RNA-loading dye, consisting of RITHOPHORESE® Sequencing gel diluent (50 %, Carl Roth, #3047.1) diluted 1:10 in H_2O supplemented with bromphenol blue sodium salt (Carl Roth, #A512.1) and xylene cyanole (Carl Roth, #A513.1). Gel was run in Tris-Boric Acid-EDTA buffer (TBE, Tris 8.9 mM, Boric Acid 8.9 mM, EDTA 0.2 mM) for about 6 h, at 65 W, 90 mA.

For visualization of bands, the gel was placed onto UV-reflecting TLC Silica gel 60 F_{254} plates (Sigma-Aldrich, #1055540001) wrapped in transparent plastic wrap and irradiated at 254 nm. guideRNAs absorbed the radiation and appeared as dark bands. guideRNAs bearing the snap/$(snap)_2$-linker run slower on the gel. As a reference, uncoupled NH_2-guideRNA was loaded in a separate well. Bands of correct size were excised, and gel slices were shaken over-night at 4°C in nuclease-free H_2O. Purification was done by ethanol precipitation. For this, 0.1 volumes of sodium acetate (3 M in H_2O) and 3.5 volumes of ethanol were added to the snap-gRNA solution and precipitated over-night at -20°C. Next, the mix was centrifuged for 60 min at 4°C with 14,000 RPM. Supernatant was discarded and pellet was washed with 500 µl of pre-chilled 70% Ethanol (in H_2O) and centrifuged again for 60 min at 4°C with 14,000 RPM. Pellet was dissolved in nuclease-free H_2O and concentration and purity were determined by NanoDrop™ 2000/2000c Spectrophotometers (ThermoFisher Scientific).

Transfection of Snap-editases expressing cells

3×10^5 HEK 293 Flp-In™ T-Rex™ cells stably expressing editases were seeded in a 24-well format in DMEM with 10% FBS supplemented with doxycycline (10 ng/ml final concentration) to induce transgene expression. After 24 h, cells were transfected with 300 ng of pcDNA 3.1 expressing transcript of interest with 0.9µl Lipofectamine™ 2000. 24 h post-transfected 8×10^4 cells were reverse transfected with 5 pmol (unless differently stated) of snap/$(snap)_2$-guideRNA and 0.75 µl Lipofectamine™ 2000 by pipetting the transfection mix in a 96-well format and dripping cell suspension onto it. Unless differently stated, editing endpoint was 48 h post guideRNA transfection. When endogenous targets were edited, the forward transfection of pDNA was omitted and gRNAs were transfected 24 h after seeding.

Editing with RESCUE-S

Cloning of guideRNA oligos

Oligos coding for the guideRNAs bearing the corresponding overhangs were cloned as described before (Abudayyeh, O. O., et al., Science 365.6451 (2019): 382-86). Multiple oligos were designed

based on reported designs or best reported designs were purchased. Oligos were annealed and phosphorylated. For this, 1 pmol of either oligo (1 µl of 100 µM in H_2O) were diluted in H_2O (200 µl final volume). 39 µl of dilution was incubated with 5 µl of ATP (10 mM, NewEngland Biolabs, #P0765S) and 10 units of T4 Polynucleotide Kinase (PNK; NewEngland Biolabs, #M0201L) in 50 µl final volume for 30 minutes at 37 °C. After that, mix was heated up to 95 °C and slowly cooled down to room-temperature to ensure correct annealing of phosphorylated oligos. 1 µg of pC0041 pDNA for gRNA expression (Addgene, #103852) was digested with *Bbs* I (NewEngland Biolabs) in 50 µl total volume for 1 h at 37 °C. Successful digest was determined by gel electrophoresis in a 1.4 % Agarose gel (ROTI®Garose Agarose NEEO ultra-quality, Carl Roth, #2267.4) in Tris-Acetate-EDTA-Buffer (TAE; 40 mM Tris, 20 mM Acetic Acid, 1 mM EDTA,) for 30 min. at 120 V. Correct bands were excised an isolated using the NucleoSpin® Gel and PCR Clean-up Mini kit (Macherey-Nagel, #740609.50) following manufacturer's instructions. 1 µl of oligo phosphorylation and annealing reaction mix was ligated into 30 ng of linearized vector using 0.5 µl of T4 DNA Ligase (NewEngland Biolabs, #M0202L) in 10 µl final volume by incubation at room temperature for 15 min. All of the mix was used for heat shock transformation of XL-1 Blue chemically competent bacteria. 50 µl of chemically competent bacteria were diluted in 100 µl of Tris-EDTA buffer (TE; 10 mM Tris-HCl, 1 mM Na-EDTA). All of the mix was added to the mix and incubated on ice for 30 min. After that, mix was incubated at 42 °C for 1 min. and further incubated on ice for 5 min. Bacteria were reactivated in 1 ml of LB-medium (25 g of LB Broth, Carl Roth, #X968.2 in 1 l of H_2O) and shaking at 37 °C for 1 h. Cells were then streaked on an Ampicillin sodium salt CELLPURE® (Amp; Carl Roth, #K029.1, 100 µg/ml final conc.) containing LB-medium plate and incubated at 37 °C over-night. Single colonies were picked and incubated in liquid LB-medium while shaking at 37 °C over-night. Plasmids were isolated from the liquid cultures by using the NucleoSpin® Plasmid Transfection-grade kit (MachereyNagel, #740490.250) following manufacturer's instructions.

Transfection of RESCUE-S cell lines

2 x 10^4 HEK 293 Flp-In™ T-Rex™ cells stably expressing RESCUE-S were seeded in a 96-well format in DMEM with 10% FBS supplemented with doxycycline (10 ng/ml final concentration) to induce

transgene expression. After 24 h cells were transfected, as previously described (Abudayyeh, O. O., et al., Science 365.6451 (2019): 382-86). Cells were transfected with 300 ng of guideRNA expressing pDNA and 40 ng of target/reporter pDNA with 0.5 µl Lipofectamine™ 2000. Editing endpoint was 48 h post guideRNA transfection. When endogenous targets were edited, target pDNA was excluded.

Analysis of editing yield

At endpoint, cells were lysed with RLT-buffer (Qiagen, #79216) and the total RNA was isolated using the Monarch® RNA Cleanup Kit 10 µg (New England BioLabs, #T2030L) following manufacturer's instructions. For DNA depletion, 1 µg of total RNA was incubated for 30 min at 37°C with DNase I (NewEngland BioLabs, #M0303L) in 25 µl total volume. Reaction was terminated by addition of 2 µl EDTA (25 mM) and incubation for 10 min. at 75 °C. 7.5 µl of reaction (~250 ng) was utilized for target site amplification using either the One Step RT-PCR Kit (BiotechRabbit, #BR0400102) or One*Taq*® One-Step RT-PCR Kit (NewEngland BioLabs, #E5315S) with the according primers in 25 µl total volume. Correct amplicon size was determined by agarose gel electrophoresis. For this, samples were loaded on a 1.4 % agarose gel in TAE (> 1,000 bp) or Sodium-Borate-Buffer (SB; 10 mM NaOH, 36.5 mM Boric Acid, <1,000 bp amplicon) and separated at 120 V for 30 min (TAE) or 200 V for 15 min (SB). Correct amplicons were excised and isolated using the NucleoSpin® Gel and PCR Clean-up Mini kit following manufacturer's instructions. 100ng of amplicon was sequenced with the respective primer. Sanger Sequencing was carried out by Microsynth or Eurofins.

Editing analysis of APOE

APOE is a high GC-content transcript. Therefore, downstream preparation had to be adjusted. Post RNA isolation, 1 µg of total RNA was DNA-depleted with TURBO DNA-free™ kit (ThermoFisher Scientific, #AM1907) in 30 µl total volume (1 µl of TURBO™ DNase) for 30 min. at 37 °C. Reaction was terminated by addition of 2 µl of Turbo DNase™ Inactivation buffer and incubation at room-temperature for 5 min. 5 µl of mix was then used for RT conducted using the

SuperScript™ IV First-Strand Synthesis System (ThermoFisher Scientific, #18091050). Mix was incubated with 1 µl of dNTPs, 2 µl of random primer mix (10x), and 1 µl of gRNA sense strand oligo (10 µM) at 95 °C for 5 min. and immediately afterwards cooled down on ice. Next, RT was set up by addition of 4 µl of SuperScript™ IV buffer (5x), 1 µl DTT (0.1 M), 1 µl RNase Inhibitor (murine), and SuperScript™ IV RT in 20 µl final volume. 5 µl of reaction mix was used for PCR with Taq Polymerase (NEB, #M0267S) containing DMSO (10% final conc.) in 50 µl final volume. Both, determination of correct amplicon size and its excision was conducted as described above. Sequencing was also performed as described above.

CTNNB1 Assay

Transfection conditions

3 x 10^5 HEK 293 FlpIn TRex cells stably expressing SNAP-CDAR-S were seeded in a 24-well format in DMEM with 10% FBS supplemented with doxycycline (10 ng/ml final concentration) to induce transgene expression. After 24 h cells were forward transfected with 300 ng of pcDNA 3.1 expressing Renilla Luciferase and 300 ng of either TOPFlash (FireFly Luciferase expression with TCF/LEF responsive promoter elements; Addgene #12456) or FOPFlash (FireFly Luciferase expression with mutated TCF/LEF responsive promoter elements; Addgene #12457) pDNA with 0.9 µl (per 300 ng pDNA) of Lipofectamine™ 2000 (Life Technologies). 24 h after pDNA transfection, 8 x 10^4 cells were either reverse transfected as described with 5 pmol of either CTNNB1 T41-targeting NH_2-gRNA, CTNNB1 T41-targeting $(snap)_2$-gRNA, PPIB R7C-targeting $(snap)_2$-gRNA or without any gRNA in technical duplicates. 48 h after gRNA transfection FireFly expression was measured with the Dual-Luciferase® Reporter Assay System by Promega according to manufacturer's instructions. Cells were lysed with 30 µl of Passive Lysis buffer per well (96-well format) and shaken for 15 min. at room temperature.

For RESCUE-S transfection, 2 x 10^4 HEK 293 Flp-In™ T-Rex™ cells stably expressing RESCUE-S were seeded in a 96-well format in DMEM with 10% FBS supplemented with doxycycline (10 ng/ml final concentration) to induce transgene expression. After 24 h cells were transfected, as previously described (Abudayyeh, O. O., et al., Science 365.6451 (2019): 382-86). Briefly, cells

were transfected with 300 ng of pDNA expressing either CTNNB1 T41-targeting, PPIB R7C-targeting or no gRNA at all, 20 ng of pcDNA3.1 expressing Renilla Luciferase and 20 ng of TOPFlash or FOPFLash pDNA with 0.5 µl Lipofectamine™ 2000 (Life Technologies) in technical quadruplicates. Another sample was included transfected only with Luciferase expressing pDNA. 48 h after gRNA transfection, FireFly expression was measured with the Dual-Luciferase® Reporter Assay System (Promega, E1910) according to manufacturer's instructions. Cells were lysed with 20 µl (per well) of Passive Lysis buffer per well (96-well format) and shaken for 15 min. at room temperature and two technical replicates were pooled.

FireFly Luciferase expression and editing yield

10 µl of each replicate was measured in a LumiNunc 96-well plate (VWR) with a Spark 10 M plate reader (Tecan). 35 µl per well of each substrate was added by an auto-injector in sequence. For FireFly Luciferase signal measurement, 35 µl per well of Luciferase Assay Reagent II (LAR II, Promega) were injected and incubated for 5 seconds, after which signal was measured for 10 seconds. For Renilla Luciferase signal measurement and FireFly Luciferase signal quenching, 35 µl per well of Stop & Glo® Reagent and incubated for 5 seconds, after which signal was measured for 10 seconds.

Technical duplicates were pooled, and total RNA was isolated using the Monarch® RNA Cleanup Kit 10 µg (New England BioLabs) following manufacturer's instructions. Editing yield was determined as described above.

FireFly signal and editing yield were determined in three biological replicates.

STAT3 Assay

3 x 10^5 HEK 293 FlpIn TRex cells stably expressing editases were seeded in a 24-well format in DMEM with 10% FBS supplemented with doxycycline (10 ng/ml final concentration) to induce transgene expression. After 24h, 3.2 x 10^5 cells were reverse transfected with 20 pmol of STAT3 S727F-targeting NH_2-gRNA, $(snap)_2$-gRNA, or PPIB R7C-targeting $(snap)_2$-gRNA (quadruple of 96-

well format) using 2 µl Lipofectamine™ RNAiMAX (invitrogen) per transfection. Transfection was repeated in the same fashion 2, 4, and 6 days after the first transfection. Endpoint was at day 8 after the first transfection. Cells were harvested and 20% were used for RNA isolation and editing analysis as described above. For cell lysis 10 ml of RIPA lysis and extraction buffer (ThermoFisher Scientific) was supplemented with one tablet of c0mplete™ Tablets, Mini, EDTA-free *EASY*pack Protease Inhibitor Cocktail (Roche) and PhosStop *EASY*pack (Roche), respectively. The rest of the cells was lysed in 50 µl of lysis buffer, incubated on ice for 15 min, and centrifuged for 10 min. at 4 °C and 14,000 RPM. Supernatant was collected and protein concentration was measured by Pierce™ BCA Protein Assay Kit (ThermFisher Scientific) in a Tecan Plate Reader (gucken wie das eigentlich heißt). 30 µg of protein were loaded in duplicates on a Novex™ WedgeWell™ 8 to 16%, Tris-Glycine, 1.0 mm, Mini Protein Gel (ThermoFisher Scientific) and separated for 60 min. at 200 V in 1:10 ROTHIPHORESE® 10X SDS-PAGE (Carl Roth, #3060):H2O. Blotting was performed with the Mini Trans-Blot Cell® (BioRad) in TransferBuffer (190 mM glycine, 25 mM Tris, 20% Methanol) for 60 min. at 100 V. Blocking was performed for 1 h at room temperature in blocking buffer consisting of Tris-buffered saline (TBS, 50 mM Tris, 150 mM NaCl) with Tween® 20 (VWR, #M147-1L, 1 % final conc.; TBST) containing Milk, non fat (skimmed milk), powder (VWR, 5 % final concentration). All antibodies were diluted in blocking buffer. The blot was cut in half and one side was used for total STAT3 detection and the other side was used for STAT3 pS727 detection. The former was incubated with Stat3 (DRZ2G) Rabbit mAb (CellSignaling) in a 1:1000 dilution and Monoclonal Anti-β-Actin antibody produced in mouse (Sigma) in a 1:5,000 dilution. The latter was incubated with P-Stat3 (S727) (D8C2Z) Rabbit mAb (CellSignaling) in a 1:1,000 dilution and Monoclonal Anti-β-Actin antibody produced in mouse (Sigma) in a 1:5,000 dilution. Incubation was performed for 3 days at 4 °C. Blots were washed three times for 5 min. with TBST. Secondary antibody incubation was performed using Peroxidase AffiniPure Goat Anti-Mouse IgG (Jackson Immuno Research, #115-035-003), and Peroxidase AffiniPure Goat Anti-Rabbit IgG (Jackson Immuno Research, #111-035-003) dissolved in blocking buffer at a 1:10,000 dilution for 90 min. at room temperature. Blots were washed again with TBST three times for 5 min. Blots were incubated with detection solution (100 mM Tris-HCl, 0.022 % Luminol, 0.0033 % p-coumaric acid) Images were taken with Odyssey FC Imager (Li-Cor® Biosciences).

Next Generation Sequencing

For Next Generation Sequencing (NGS), samples transfected without anything or with PPIB R7C-targeting gRNA (2.5 pmol)/gRNA expression vector (300 ng) in technical duplicates. For this, cells expressing editases were transfected in a five-fold set up, as described above. 48 h after transfection, cells were lysed with 50 µl per well of RLT buffer (Qiagen) and corresponding wells were pooled (250 µl total volume). 200 µl of RLT buffer was added and the RNA was isolated using the RNeasy MinElute Cleanup Kit (Qiagen, #74204) following manufacturer's instructions for large scale. RNA was eluted in 30 µl of nuclease-free H_2O and concentration was determined by Nanodrop. Sasmples were DNA-depleted by DNase I (NewEngland BioLabs) digestion. For this, entire RNA was incubated with DNase I (1 µl per 2 µg of total RNA) for 30 min. at 37 °C. RNA was then purified using the RNeasy MinElute Cleanup Kit following manufacturer's instructions for small scale. RNA was then eluted in 30 µl of nucleas-free H_2O. Concentration was adjusted to 150 ng/µl and determined by Nanodrop in duplicate. Successful execution of editing was determined by Sanger Sequencing. 500 ng of total RNA was used for amplification of target site by One*Taq*® One-Step RT-PCR Kit (NewEngland BioLabs) following manufacturer's instructions. Sample preparation for sequencing was performed as described above. The samples were submitted in their entirety for NGS performed by CeGaT. Library preparation was conducted with 100 ng of RNA with the TruSeq Stranded mRNA (Illumina). Sequencing was performed 2 x 100 bp pair-end with NovaSeq6000 (Illumina) with 50 Mio reads. Lanes of raw RNA sequencing data of same samples were pulled together, and adapter sequences were trimmed with Trim Galore (v0.6.5; http://www.bioinformatics.babraham.ac.uk/projects/trim_galore/). Sequencing alignment to the human reference genome (hg19) was performed using STAR (v. 2.7.10a). hg19 and the RefSeq annotation are publicly available at the genome browser at UCSC. For alignment uniquely mapped (STAR option: --outFilterMultimapNmax 1) reads were considered to prevent multimapping of regions of high similarity. Next, data (bam files) were deduplicated, sorted, and indexed using SAMtools (v1.9; http://samtools.sourceforge.net). SNVs calling was performed with REDItools (v2; https://github.com/tflati/reditools2.0). Developers' recommendations were taken into consideration for preceding data preparation. As previously performed, only high-

quality sites (min. MeanQ > 30 in REDItools2) were considered. Editing site were called when well-covered i.e., minimum 50 reads (in summary of duplicates per sample), showing ≥ 5 % editing frequency compared to the control. For sites matching criteria, fisher's exact test was performed. Significance was defined for all samples showing an adjusted *p*-value < 0.01. Genomic coordinates were annotated with Variant Effect Predictor (VEP) (v102) using the following command line:

```
vep –input file input..txt --fasta reference.fa --output_file output.txt --species homo_sapiens --tab --cache --dir_cache ../Human/dir --no_check_variants_order --transcript_version --canonical --ccds --hgvs --symbol --gene_phenotype --pubmed --variant_class --pick --offline --force_overwrite
```

Supplementary Figures

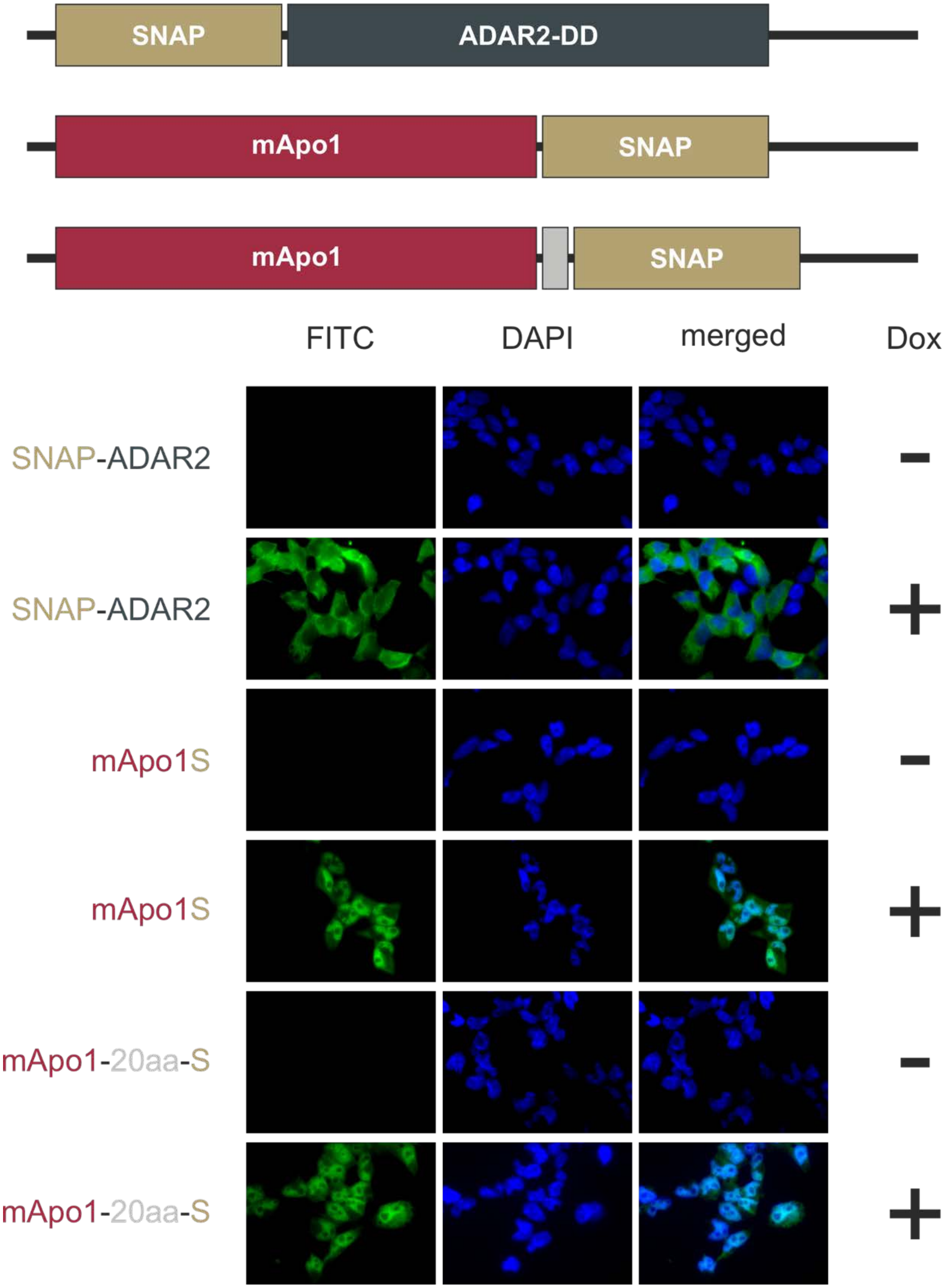

S. Figure S1: Microscopy of SNAP-tagged effectors. 5 x 10^4 cells were plated on glass cover slips and SNAP-effector expression was induced for 24 h with doxycycline. Images were taken at 24 h after doxycycline induction. SNAP-effectors were visualized by incubation with BG-FITC and DAPI stain was utilized for nucleus visualization. SNAP-ADAR2 served as control for cytoplasmatic localization.

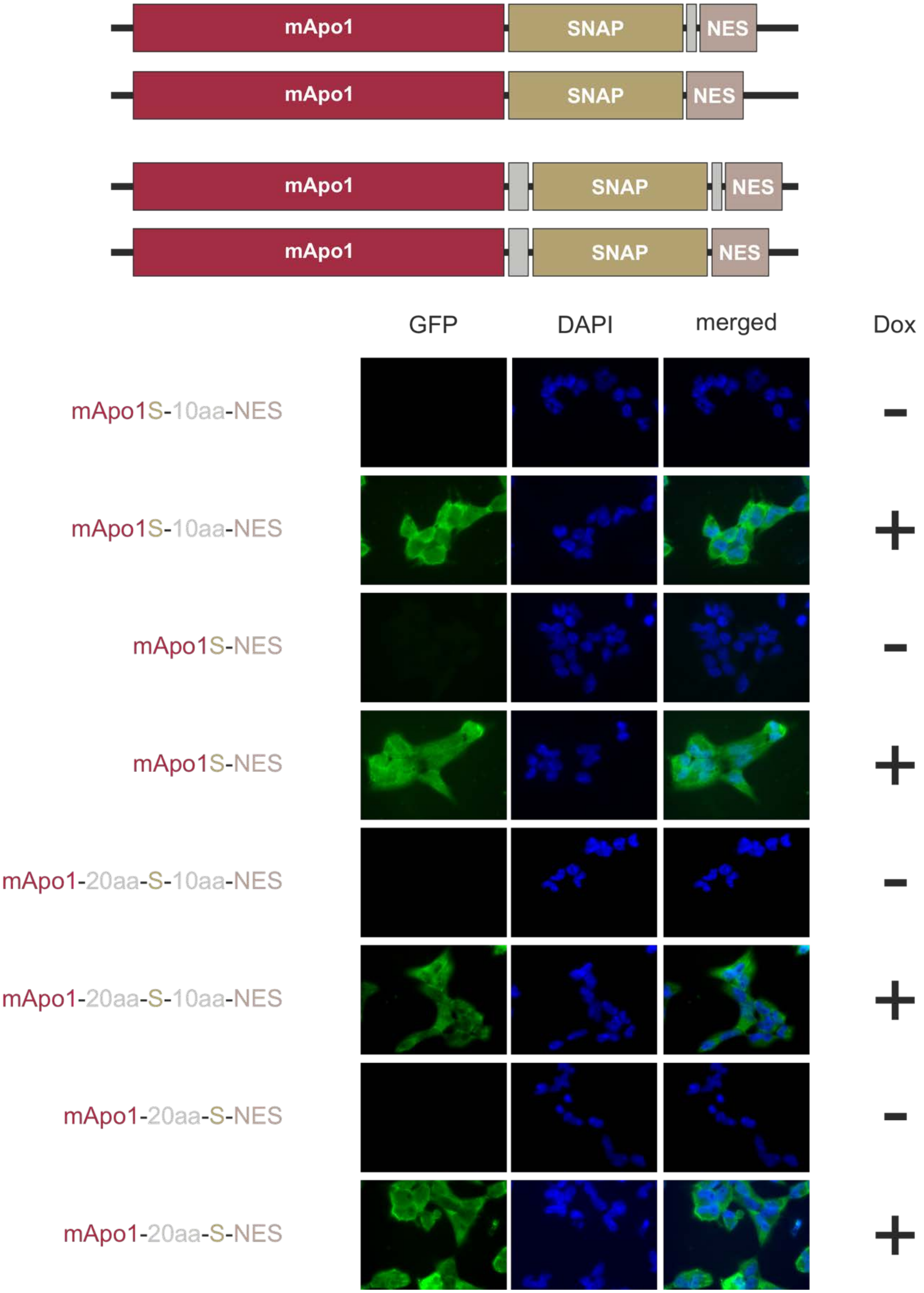

S. Figure S2: Microscopy of mApo1S constructs bearing a nuclear export signal (NES) of the HIV Rev protein. 5 x 10^4 cells were plated on glass cover slips and SNAP-effector expression was induced for 24 h with doxycycline. Images were taken at 24 h after doxycycline induction. SNAP-effectors were visualized by incubation with BG-FITC and DAPI stain was utilized for nucleus visualization.

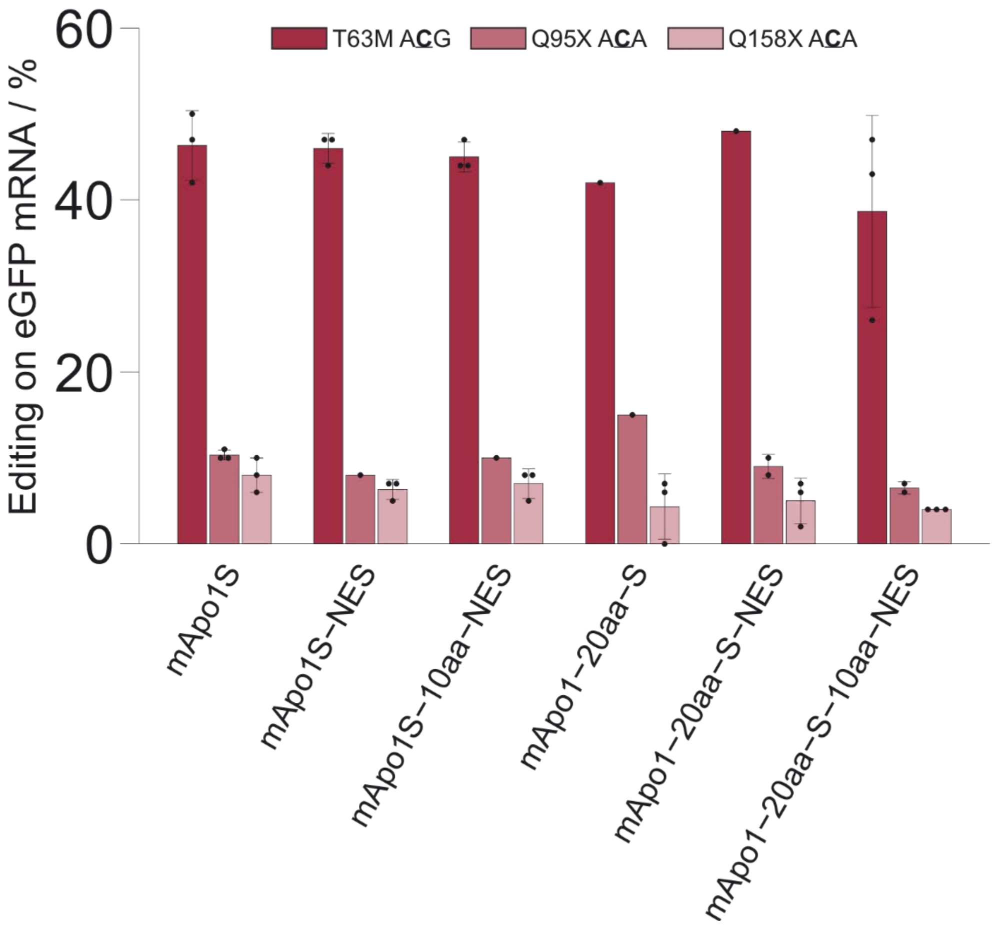

S. Figure S3: Effect of localization tags on Editing. 8 x 10^4 Cells stably expressing mApo1S constructs were transfected with 5 pmol of $(snap)_2$-gRNA targeting eGFP T63, Q95, or Q158, respectively. Editing yield was determined 24 h post-transfection. Data is shown as mean ± s.d. of N ≥ 1 of independent experiments, as indicated by the individual data points.

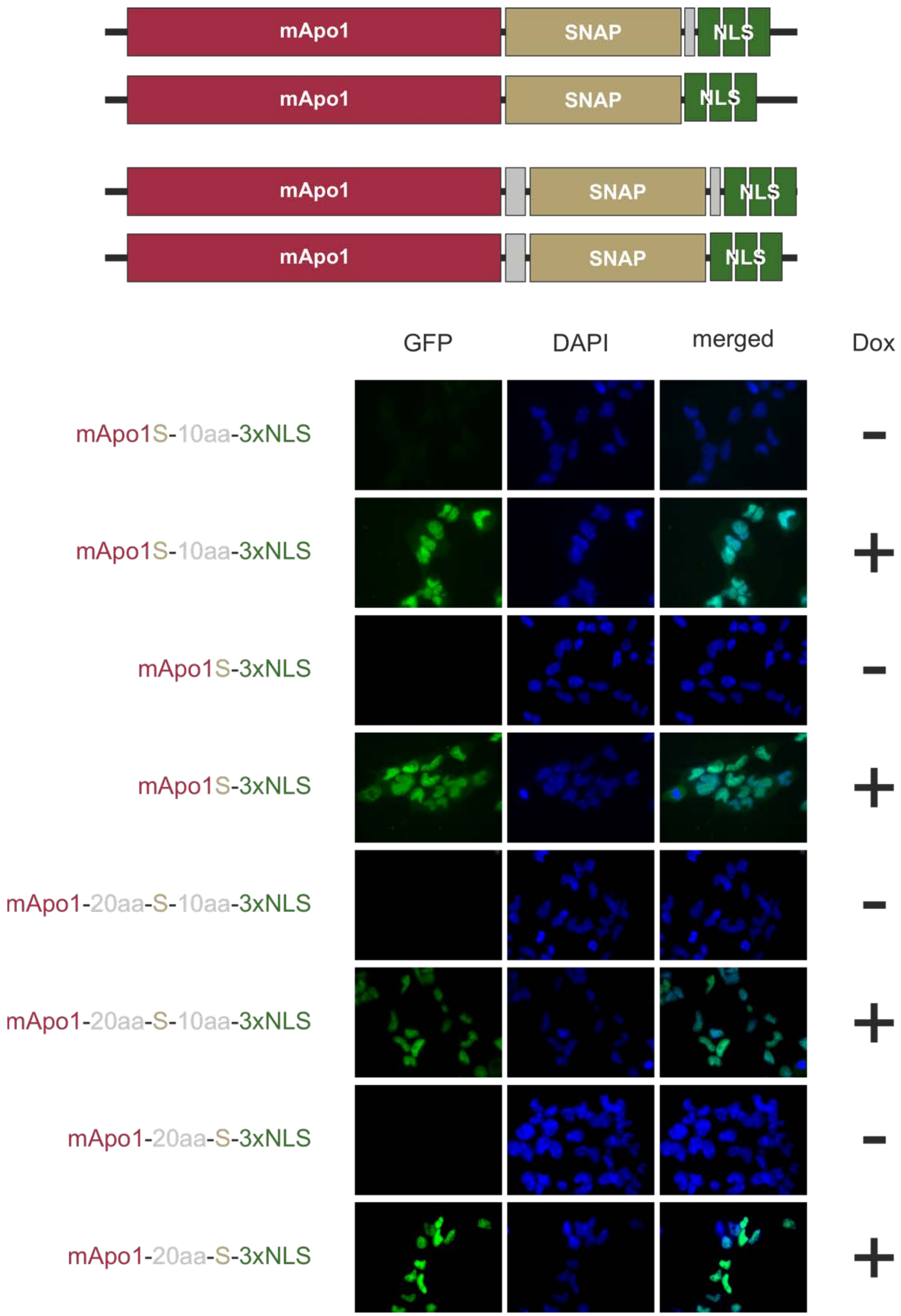

S. Figure S4: Microscopy of mApo1S constructs bearing three copies of nuclear localization signals of the SV40 large T antigen. Microscopy of mApo1S constructs bearing a nuclear export signal (NES) of the HIV Rev protein. 5 x 10^4 cells were plated on glass cover slips and SNAP-effector expression was induced for 24 h with doxycycline. Images were taken at 24 h after doxycycline induction. SNAP-effectors were visualized by incubation with BG-FITC and DAPI stain was utilized for nucleus visualization.

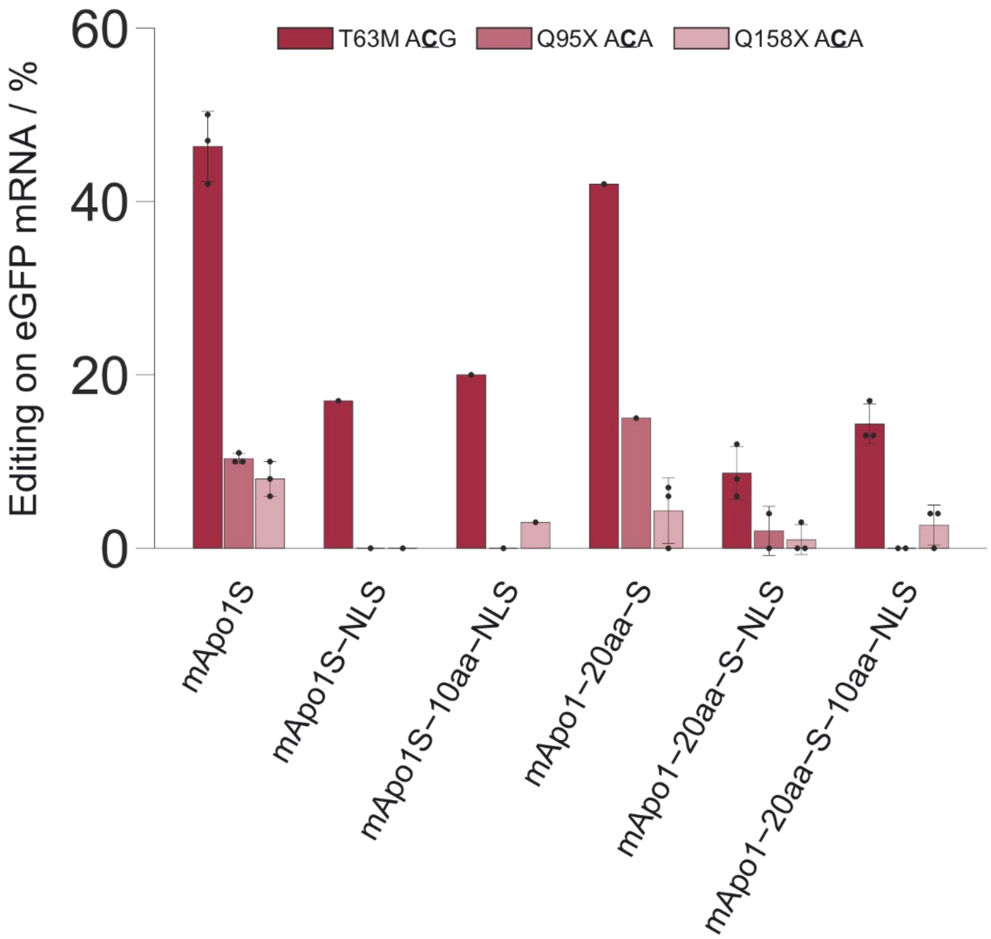

S. Figure S5: Effect of localization tags on Editing. 8 x 10^4 Cells stably expressing mApo1S constructs were transfected with 5 pmol of $(snap)_2$-gRNA targeting eGFP T63, Q95, or Q158, respectively. Editing yield was determined 24 h post-transfection. Data is shown as mean ± s.d. of N ≥ 1 of independent experiments as indicated by individual data points.

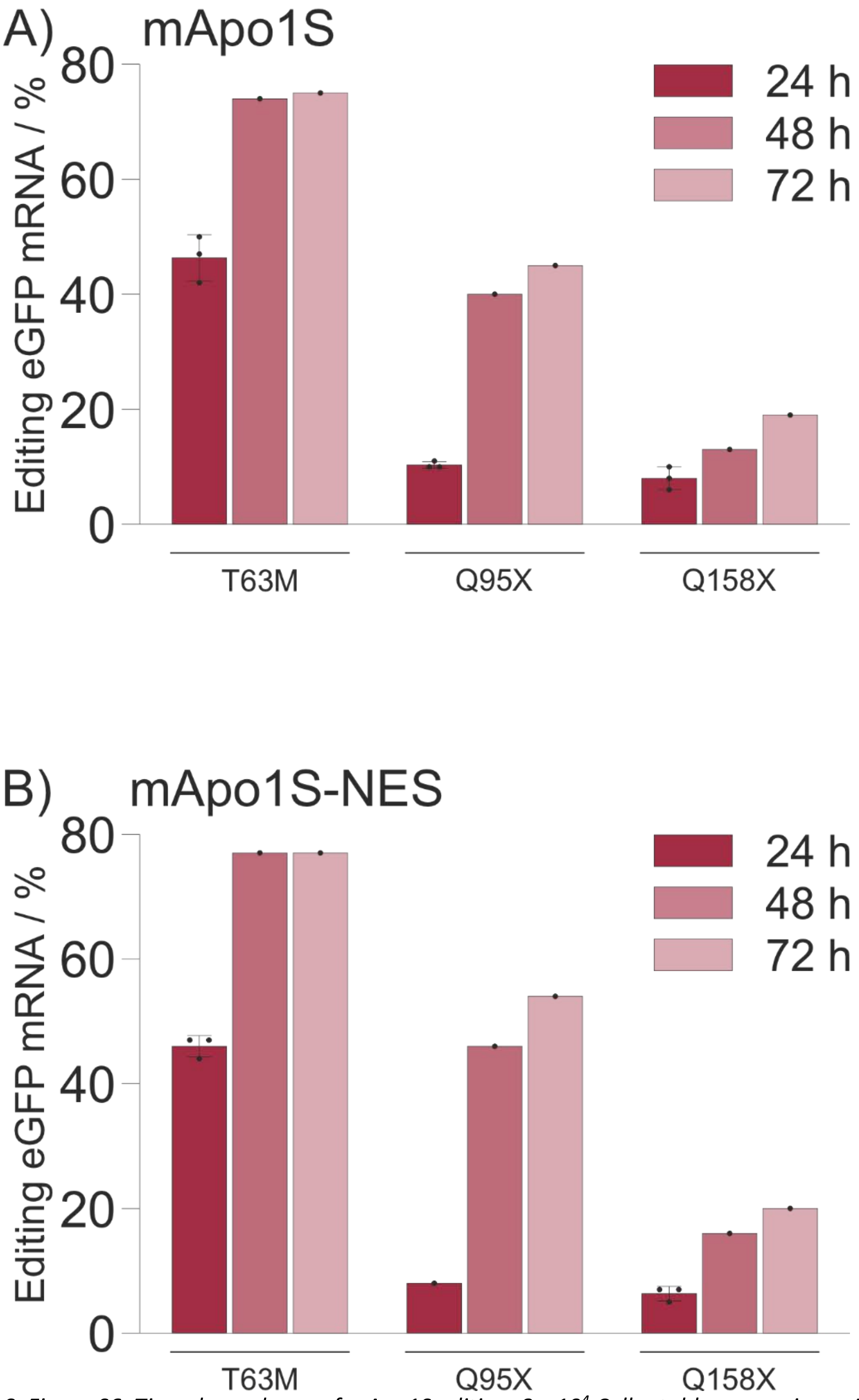

S. Figure S6: Time dependence of mApo1S editing. 8 x 10^4 Cells stably expressing mApo1S (A) or mApo1s-NES (B) were transfected with 5 pmol of $(snap)_2$-gRNA targeting eGFP T63, Q95, or Q158, respectively. Editing yield was determined at indicated time points post-transfection. Data is shown as mean ± s.d. of N ≥ 1 of independent experiments, as indicated by individual data points.

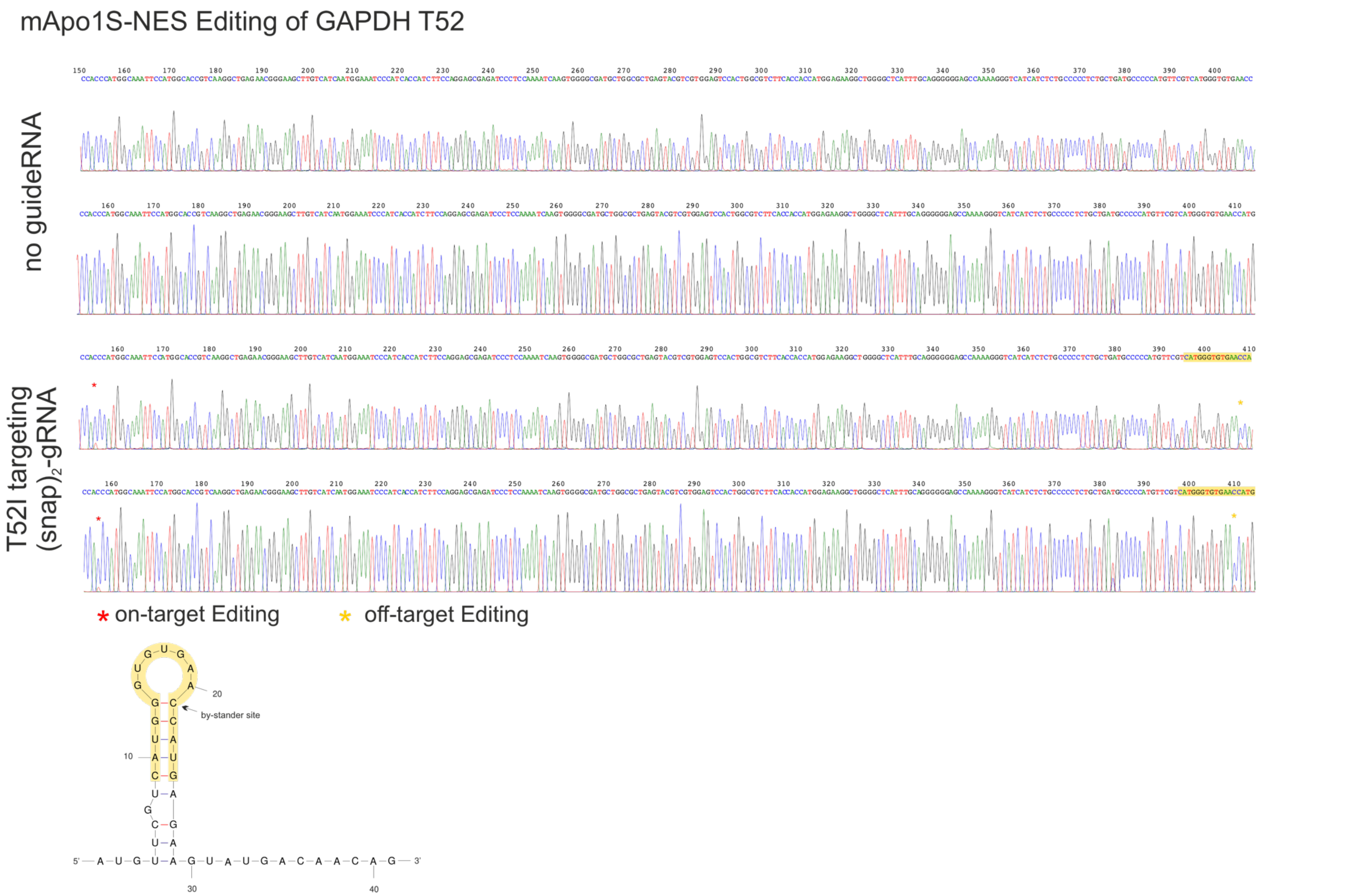

S. Figure S7: mApo1S editing of GAPDH T52. 8 x 10[4] cells were transfected with 5 pmol of (snap)$_2$-gRNA targeting GAPDH T52. Endpoint was 48 h post-transfection. On-target editing is shown in duplicates of independent experiments by red asterisk. Yellow asterisk indicates a persistent guideRNA-dependent by-stander editing, which almost exceeds the on-target editing yield and seems to be promoted by a local RNA stem-loop hairpin structure

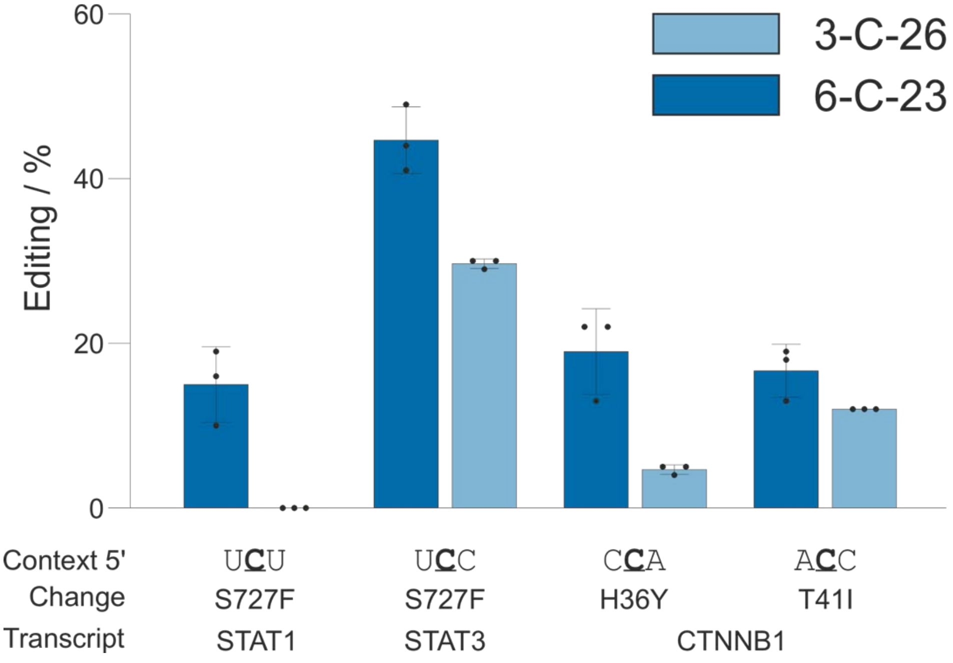

S. Figure S8: Comparison of gRNA designs. 8 x 10^4 cells stably expressing SNAP-CDAR-S were transfected with 5 pmol of (snap)$_2$-gRNA of indicated design, respectively. Best performing guideRNA design on PPIB (3-C-26) was compared to 6-C-23 design on various endogenous transcripts, indicating that the 6-C-23 design works typically better, with PPIB as the exception. Data is shown as mean ± s.d. of N = 3 of independent experiments.

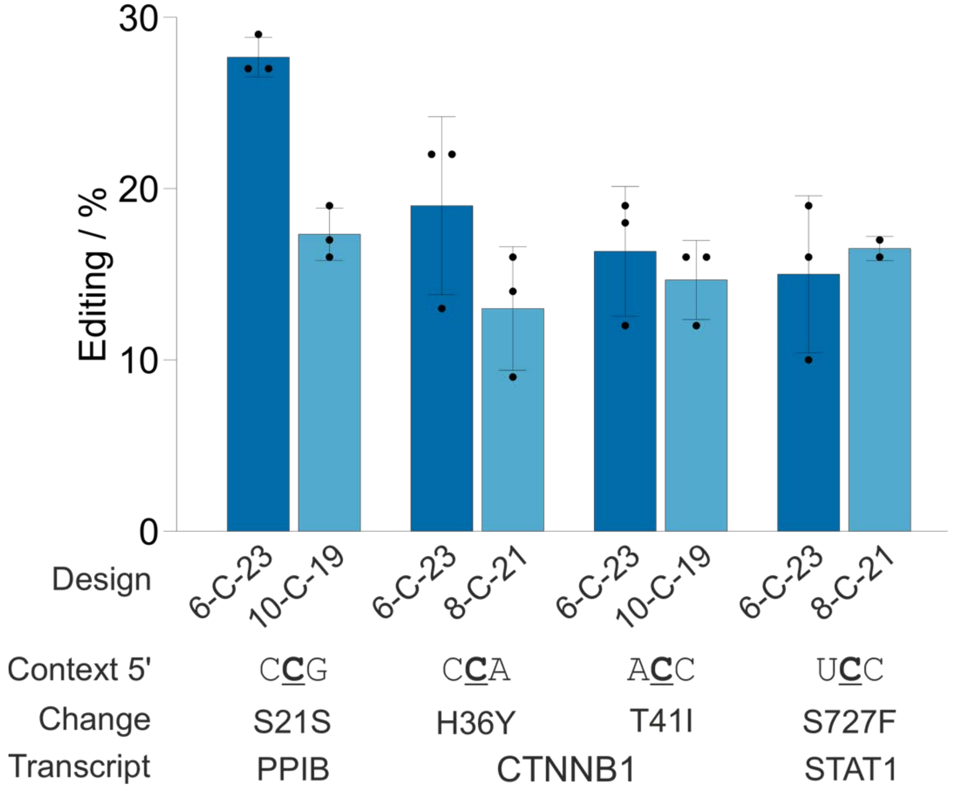

S. Figure S9: Comparison of lead gRNA designs with best reported design. 8 x 10^4 cells stably expressing SNAP-CDAR-S were transfected with 5 pmol of $(snap)_2$-gRNA of indicated design, respectively. SNAP-CDAR-S lead design (6-C-23) was compared to $(snap)_2$-gRNAs with designs reported best for RESCUE-S (position of mismatch C). Data is shown as mean ± s.d. of N = 3 of independent experiments.

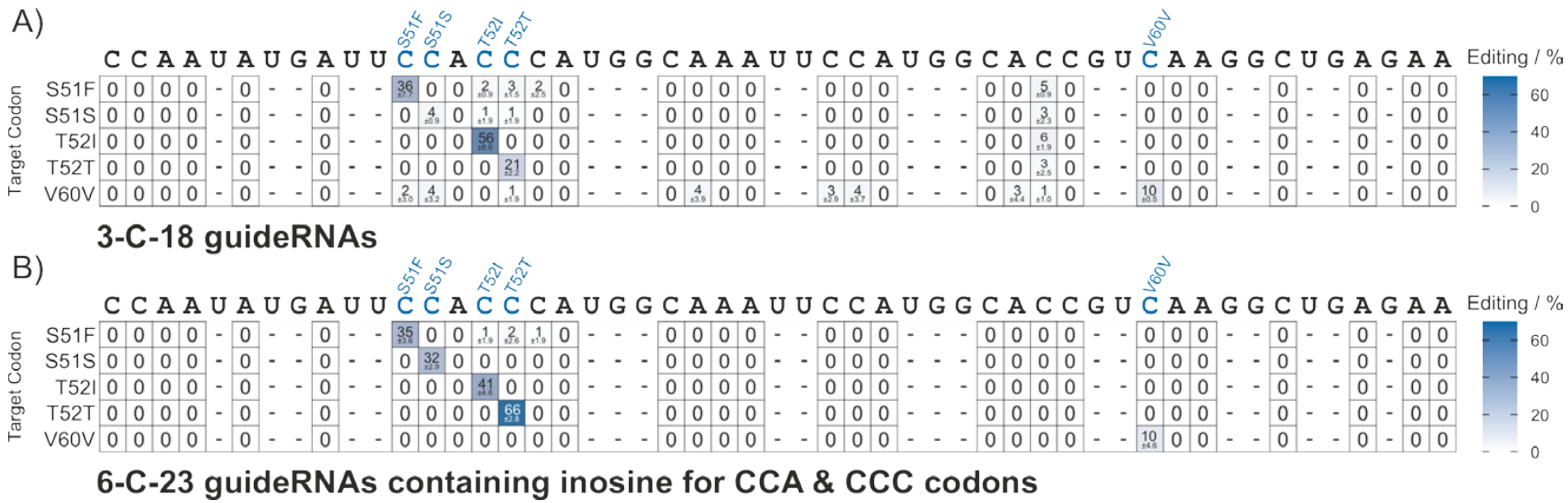

S. Figure S10: Heatmap for Programmability of SNAP-CDAR-S. 8 x 10^4 cells stably expressing SNAP-CDAR-S were transfected with 5 pmol of either 3-C-18 (A) or 6-C-23 (B) $(snap)_2$-gRNAs targeting indicated target sites on GAPDH transcript. B) gRNAs targeting 5'CCN codons contained inosine opposite of 5'C. Depicted are a 51 bp area of GPADH transcript surrounding the target sites. Increase of on-target editing by the 6-C-23 design did not result in reduction of fidelity, as editing of by-stander sites remained at background level. Data is shown as mean ± s.d. of N = 3 of independent experiments.

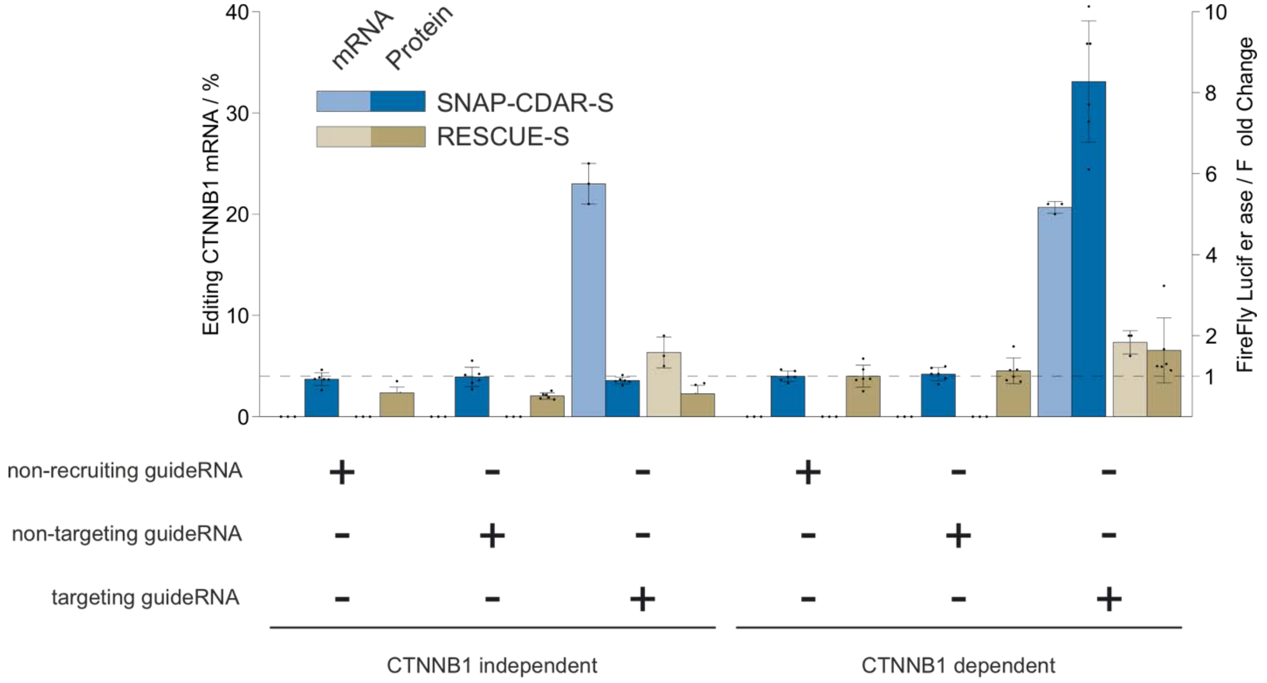

S. Figure S11: FireFly Luciferase Assay of CTNNB1 T41 Editing. FireFly Luciferase signal as fold change of cells transfected with a CTNNB1-dependent reporter plasmid DNA and a non-recruiting guideRNA. Definition of "non-recruiting guideRNA" was system-dependent (i.e., CTNNB1 T41-targeting NH_2-guideRNA for SNAP-CDAR-S and empty guideRNA expression plasmid for RESCUE-S). Data is shown as mean ± s.d. of N = 3 independent experiments. FireFly Luciferase signal was measured in technical duplicates of independent experiments, respectively.

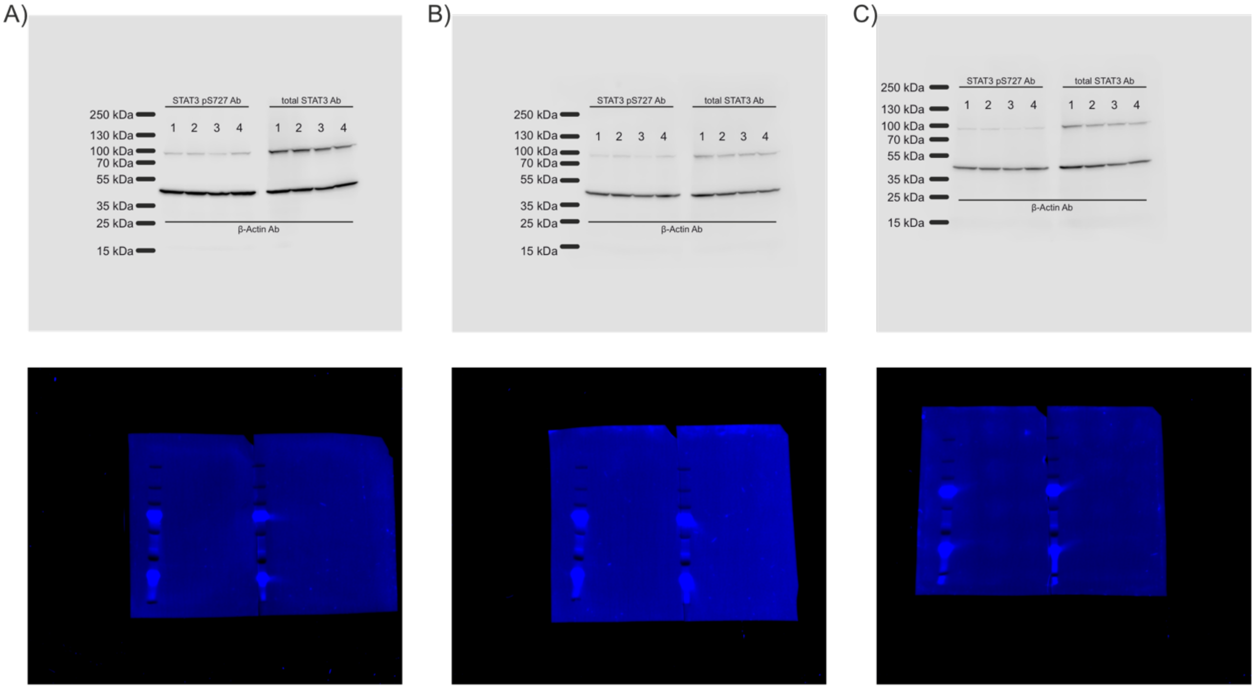

Legend

1: no guideRNA

2: STAT3 S727F NH_2-guideRNA

3: STAT3 S727F $(snap)_2$-guideRNA

4: PPIB R7C $(snap)_2$-guideRNA

S. Figure S12: STAT3 pS727 Editing. SNAP-CDAR-S stably expressing cells were transfected every 48 h with 5 pmol of (snap)2-gRNA targeting STAT3 S727 over a time period of 8 days. At endpoint, 30 µg of protein lysate was applied in duplicates. Lower row: Visualization of protein marker. Upper row: Visualization of phosphorylated S727 of STAT3 (left part of gels) and total STAT3 (right part of gels). The experiment was done in a triplicate of three independent experiments (A-C).

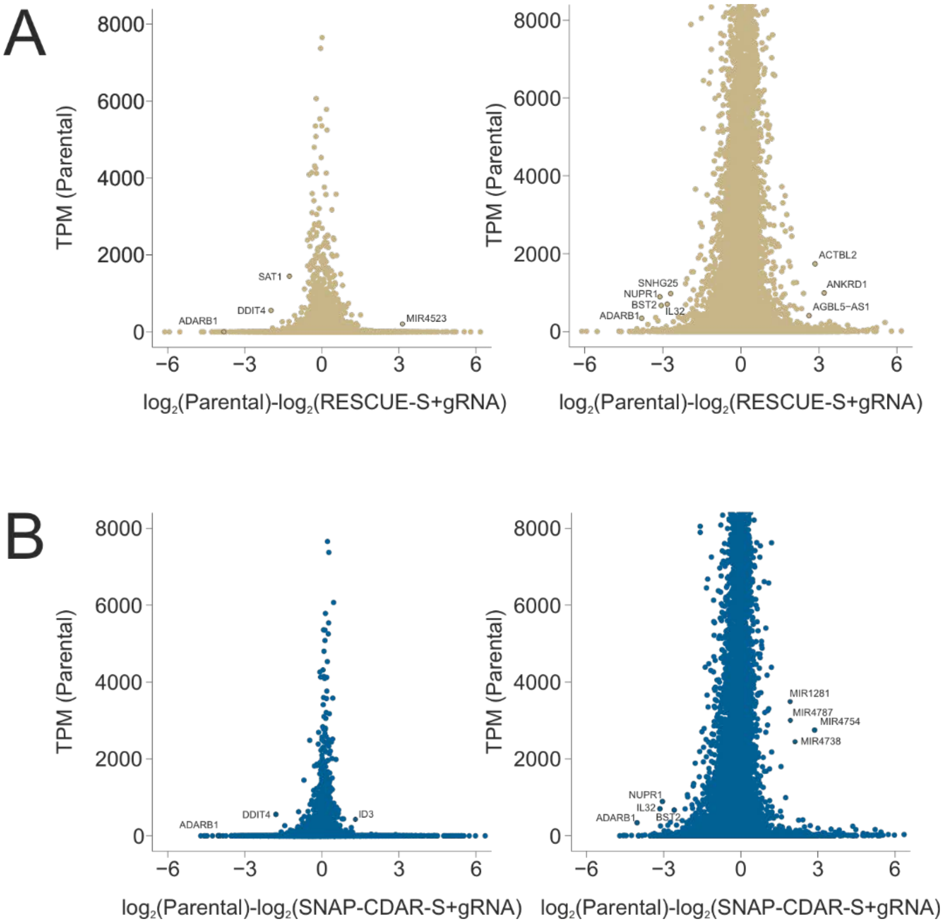

S. Figure S13: Volcano Plots of differential TPM-values of transcribed RNAs. A) RESCUE-S expressing cell lines. B) SNAP-CDAR-S expressing cell lines.

A

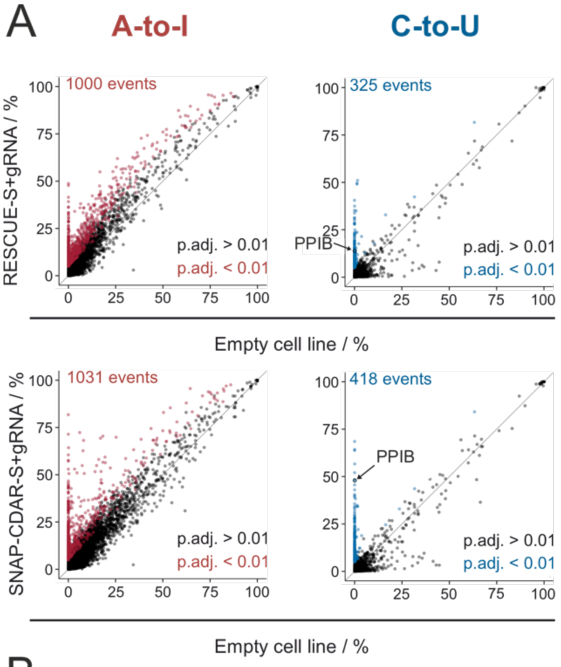

Empty cell line vs. Enzyme (+gRNA)	RESCUE-S A-to-I	RESCUE-S C-to-U	SNAP-CDAR-S A-to-I	SNAP-CDAR-S C-to-U
5'UTR	12	14	10	19
non-coding	201	26	203	53
non coding RNA	9	4	15	7
missense	192	82	178	83
stop lost	3	0	7	0
stop gained	0	16	0	13
silent	89	63	106	62
3'UTR	494	120	512	181
**total**	**1000**	**325**	**1031**	**418**

B

Table 1 | Global off-target editing

Enzyme[a]	Total	Known Alu	Known Non-Alu	Novel Alu	Novel Non-Alu	Location in mRNA 5′-UTR	Coding region Syn.	Coding region Nonsyn.	3′-UTR	Others[b]
SA1	6	2	1	0	3	0	0	1	3	2
SA2	30	15	8	1	6	0	0	2	22	6
SA1Q	835	70	59	7	699	11	117	230	402	75
SA2Q	1,310	267	71	24	948	13	149	347	637	164

Numbers represent the number of sites that were significantly differently edited compared with sites in a related control cell line that did not express the respective SA editase. Syn., synonymous; nonsyn., nonsynonymous. [a]Editing was carried out in cells expressing the given SNAP-ADAR in the presence of a BG-gRNA targeting the *ACTB* transcript. [b]"Others" refers to editing in introns, intergenic regions, and noncoding RNA.

S. Figure S14: Comparison of transcriptome-wide off-target sites. A) A-to-I and C-to-U transcriptome-wide off-target sites caused by SNAP-CDAR-S or Cas13-based RESCUE-S under stable integration of the respective effector into 293 Flp-In T-REx cell lines. B) For comparison, characterization of transcriptome-wide A-to-I off-target sites caused by stable integration of different SNAP-ADAR effectors in 293 Flp-In T-REx cell lines (SA1: wildtype SNAP-ADAR1; SA2: wildtype SNAP-ADAR2; SA1Q: hyperactive SNAP-ADAR1Q; SA2Q: hyperactive SNAP-ADAR2Q). The data indicates that the RESCUE-S domain, evolved form ADAR2Q, has still considerable A-to-I off-target effects, clearly above that of the wildtype ADAR2 deaminase. Table was taken from original publication by Paul Vogel et al. Nature Methods 2018.
